# Detransition and Desistance Among Previously Trans-Identified Young Adults

**DOI:** 10.1007/s10508-023-02716-1

**Published:** 2023-12-01

**Authors:** Lisa Littman, Stella O’Malley, Helena Kerschner, J. Michael Bailey

**Affiliations:** 1 The Institute for Comprehensive Gender Dysphoria Research, 11 S. Angell Street, #331, Providence, RI 02906 USA; 2 Cincinnati, OH USA; 3 Department of Psychology, Northwestern University, Evanston, IL USA

**Keywords:** Detransition, Desistance, Gender dysphoria, Transgender, Rapid-onset gender dysphoria, DSM-5

## Abstract

Persons who have renounced a prior transgender identification, often after some degree of social and medical transition, are increasingly visible. We recruited 78 US individuals ages 18–33 years who previously identified as transgender and had stopped identifying as transgender at least six months prior. On average, participants first identified as transgender at 17.1 years of age and had done so for 5.4 years at the time of their participation. Most (83%) participants had taken several steps toward social transition and 68% had taken at least one medical step. By retrospective reports, fewer than 17% of participants met DSM-5 diagnostic criteria for Gender Dysphoria in Childhood. In contrast, 53% of participants believed that “rapid-onset gender dysphoria” applied to them. Participants reported a high rate of psychiatric diagnoses, with many of these prior to trans-identification. Most participants (*N* = 71, 91%) were natal females. Females (43%) were more likely than males (0%) to be exclusively homosexual. Participants reported that their psychological health had improved dramatically since detransition/desistance, with marked decreases in self-harm and gender dysphoria and marked increases in flourishing. The most common reason given for initial trans-identification was confusing mental health issues or reactions to trauma for gender dysphoria. Reasons for detransition were more likely to reflect internal changes (e.g., the participants’ own thought processes) than external pressures (e.g., pressure from family). Results suggest that, for some transgender individuals, detransition is both possible and beneficial.

**Supplementary Information:**

The online version contains supplementary material available at 10.1007/s10508-023-02716-1.

## Introduction

Persons who have renounced a prior transgender identification have become increasingly visible during the past decade (Littman, 2021). Often these individuals have changed their minds after taking steps toward social and medical gender transition and may be referred to as “detransitioners.” Detransitioner communities have emerged online (e.g., r/detrans, 2019, 2020); hundreds of detransitioner testimonies can be found on YouTube and other social media platforms, in online blogs, book chapters, and in published articles (Callahan, 2018; Entwistle, 2021; twitter.com/ftmdetransed and twitter.com/radfemjourney, 2019; YouTube, 2022); and detransitioners have organized to bring awareness to their experiences and advocate for their needs (e.g., Detrans Voices, 2022; Gender Care Consumer Advocacy Network, 2022; Pique Resilience Project, 2019; Post Trans, 2022). Detransition has received attention from prominent bloggers and journalists (4thwavenow, 2016; Anonymous, 2017; Boyce, 2021; Herzog, 2017; Tracey, 2020; upperhandMARS, 2020) and even from mainstream media (McCann, 2017; Smith, 2021). This publicity has been heightened by cases in which detransitioners appear to have received inadequate oversight before they were provided serious medical interventions, such as the lawsuit filed by Kiera Bell (Topping, 2020). Clinicians and researchers have documented a growing number of detransitioners seeking psychological and medical support (D’Angelo et al., 2021; Marchiano, 2020; Vandenbussche, 2022). Because of both controversy and recency regarding detransitioners, little is known about them (Valdes & MacKinnon, 2023).

It is important to distinguish several terms common in both scientific literature and lay parlance, and to clarify how we use them. Gender dysphoria is discomfort with one’s current gender (most often the same as one’s sex at birth), regardless of the causes or manifestation of the discomfort. Transgender identification represents the commitment that one’s “true gender” is not aligned with one’s birth sex. Felt “true gender” may be opposite one’s birth sex or some other gender (e.g., nonbinary). Gender transition includes both social and medical steps taken to align one’s overall presentation with one’s felt “true gender,” typically after some period of transgender identification. Social steps can include changes in dress and appearance, name, and posture/movement. Medical steps can include cross-sex hormones and surgery. Detransition is the reversal of gender transition for any reason, although for many it includes abandonment of transgender identification. Detransition may be preceded or accompanied by the feeling that one regrets gender transition (“regrets”). Desistance refers to the waning of gender dysphoria prior to medical gender transition.

This article reports on a sample of young adults who had identified as transgender but changed their minds, most of whom had taken steps toward social and medical transition. We hoped to illuminate aspects of their gender dysphoria and gender transition, as well as their detransition and (for most) the resolution of their gender dysphoria. Before reporting our study, we provide some context in the scientific literature and the broader culture.

### Controversies About Detransition and Desistance

At least three main issues have been especially controversial regarding detransition and desistance: their frequencies, the motivations of detransitioners and desisters, and the possibility that a recent phenomenon called rapid-onset gender dysphoria (ROGD; Littman, 2018) may disproportionately contribute to both phenomena. We review these controversies below, focusing on the limited empirical evidence.

#### Prevalence of Detransition and Regret

Advocates for gender transition have tended to assert that detransition is rare (e.g., Knox, 2019; Stonewall, 2019). Much of the published data used to estimate detransition prevalence come from studies of sex-reassignment-surgery outcomes. In general, these studies have found post-surgery regret to be low (Dhejne et al., 2011; Lawrence, 2003, 2006; Pfäfflin, 1993; van de Grift et al., 2017; Wiepjes et al., 2018). Similarly, one prospective study examined regret of hormonal treatment among 55 young transgender adults who had undertaken puberty suppression and then cross-sex hormones and found no evidence of regret (de Vries et al., 2014). However, a recent study found that approximately 30% of transgender adolescents and adults discontinued cross-sex hormone treatment within four years after commencing treatment (Roberts et al., 2022).

Research reviewed so far has focused on patients who were treated prior to the recent dramatic surge of gender dysphoria in the West that has occurred during the past 15–20 years (Aitken et al, 2015). This surge has been associated with changing demographics—especially an increase among adolescent females (Zucker, 2019). It is plausible that the recent and older cohorts differ in their detransition rates. To our knowledge, only two studies have explored the prevalence of detransition in recent clinical samples. The first study, a retrospective case-note review, identified a detransition rate of 6.9% from the 175 adult patients consecutively discharged from a national Gender Identity Clinic in the UK (Hall et al., 2021). The second study audited the data from 68 patients with a diagnosis of gender dysphoria from a primary care population in the UK. Of the 41 patients who began hormonal treatments, 20% stopped taking them, and 9.8% were categorized as detransitioning (Boyd et al., 2022).

Empirical problems preclude accurately estimating the prevalence of detransitioners outside of a few settings. Studies of transition regret have been small and have not used consistent outcome indices. Importantly, detransitioned patients are especially likely to be lost to follow-up.

#### Motivations for Detransition and Desistance

An important distinction is between “core” and “non-core” detransition (Exposito-Campos, 2021). In core detransition, an individual stops identifying as transgender due to an internal shift in how they conceive of themselves. In contrast, non-core detransition is not motivated by internal doubts, but by external stressors such as transgender-related discrimination, family pressure, and financial or health barriers to gender related medical treatments (e.g., hormone replacement therapy). Although all varieties of desistance and detransition warrant further attention, core detransition has been especially controversial. Individuals who mistakenly view themselves as transgender, or who decide they are no longer transgender, may be unnecessarily burdened with harmful consequences of irreversible hormonal and surgical interventions. This is especially concerning because the fastest growing subgroup of gender dysphoric individuals seeking medical treatment comprises adolescents and young adults (Aitken et al., 2015; Zucker & Aitken, 2019).

Three recent studies using convenience samples explored reasons for detransition. Littman (2021) recruited 100 individuals (69% natal females) who had medically or surgically detransitioned, regardless of current gender identification. The most common reason participants gave for detransition (60% of participants) was that they had become more comfortable with their natal sex. Other reasons included: medical concerns (49%); the belief that gender dysphoria was an expression of other problems (e.g., trauma or mental illness; 39%); the belief that gender dysphoria was caused by participants’ inability to accept their own homosexual feelings (23%); and the experience of discrimination as trans persons (23%). The majority (55%) believed they had been inadequately evaluated, medically or psychologically, before they transitioned.

A second study recruited male and female detransitioners using the question, “Did you transition medically and/or socially and then stopped?” (Vandenbussche, 2022). Of the 237 participants, 92% were natal females. Reasons endorsed for detransition overlapped considerably with those in Littman’s (2021) study. For example, the most frequently endorsed reason for detransition was that gender dysphoria “was related to other issues” (70%), followed by “health concerns” (62%). Other common reasons included feeling that transition did not help (50%), finding other ways to deal with gender dysphoria (45%), disliking the social changes accompanying transition (44%), and experiencing a change in “political views” (43%). “Resolution of gender dysphoria” was endorsed by 15% of Littman’s subjects and by 34% of Vandenbussche’s. Only 10% of this sample endorsed “discrimination” as a reason for detransition.

The third study differed substantially in both method and results from the other two reviewed in this section. Turban et al. (2021) analyzed data from a survey of 27,715 “transgender and gender diverse” adults that included several questions about detransition. Participants were recruited “through community outreach organizations” for a survey advertised as being “for all trans people age 16 and up” (https://www.ustranssurvey.org). Thus, persons no longer identifying as transgender would be excluded. Instead, currently transgender persons were asked the following questions: “Have you ever de-transitioned? In other words, have you ever gone back to living as your sex assigned at birth, at least for a while?” This study also differed from the other two in finding among detransitioners a slight majority of natal males (55%) rather than a large majority of natal females. Finally, and in contrast to the other studies, participants categorized as having detransitioned overwhelmingly endorsed “external” (82.5%) rather than “internal” reasons (15.9%) for detransition. External factors included social pressure such as “pressure from family and societal stigma.” Internal factors included “fluctuations in or uncertainty regarding gender identity.”

#### Gender Dysphoria Typology and Detransition/Desistance

At least three types of gender dysphoria have been proposed in the clinical and research literature, although no taxonomy of gender dysphoria is universally accepted at present (Bailey & Blanchard, 2017; Zucker, 2019). Childhood-onset gender dysphoria occurs in both natal males and natal females. It typically begins early in childhood and is associated with both extreme childhood gender nonconformity and adult homosexuality. Autogynephilic gender dysphoria affects only males and is associated with autogynephilia, a natal male’s sexual arousal by imitating females (especially by cross-dressing) or imagining himself as a female. Both childhood-onset gender dysphoria and autogynephilic gender dysphoria have been studied for several decades (Blanchard, 1989; Zucker, 2005; Zucker & Bradley, 1995).

In contrast, the third kind of gender dysphoria, ROGD, was unknown until recently (approximately the past decade) (Littman, 2018). Because ROGD is a new and controversial idea, there has been little empirical research on it. The limited research conducted so far (e.g., Diaz & Bailey, 2023a, b; Littman, 2018) is consistent with the following conceptualization: Adolescents and young adults without a childhood history of gender dysphoria and often with preexisting emotional problems come to believe that they have gender dysphoria. This belief typically progresses rapidly to adoption of transgender identity and the conviction that gender transition is urgent. ROGD is facilitated by social contagion, evidenced by the common occurrence of multiple affected youths in the same peer group. The syndrome appears to be especially common among natal females, who comprise approximately 75–80% of potential cases studied so far. ROGD may in some cases represent the confusion of underlying emotional and developmental difficulties as gender dysphoria. Finally, the surge of gender dysphoria cases during the past decade is plausibly due to ROGD, although this possibility is highly contentious (Ashley, 2020; French National Academy of Medicine, 2022; Shrier, 2020; WPATH, 2018).

The literature on treatment regret has focused on persons who likely experienced either childhood-onset or autogynephilic gender dysphoria. This is because these persons were treated before ROGD was noticed, and perhaps before ROGD existed at detectable levels. Thus, the generally positive results of these studies (i.e., low rates of regret) may not apply to those fitting the ROGD profile. Indeed, if ROGD is due to the misattribution of emotional and developmental difficulties to an underlying transgender status, these cases may have especially high rates of regret.

To study detransition/desistance across the different types of gender dysphoria, it is necessary to distinguish the different types accurately. Childhood-onset gender dysphoria is relatively easy to diagnose during childhood. After then, however, assessment relies on retrospective reports, which can be inaccurate for various reasons, including memory limitations and motivated distortion, especially exaggeration of childhood signs of gender dysphoria (Lawrence, 2012; Littman, 2018). An additional complication is distinguishing between natal males with autogynephilic gender dysphoria and natal males whose gender dysphoria results from ROGD, as we have described it. To family members and friends, autogynephilic gender dysphoria may appear sudden, because the autogynephilic person has probably neither appeared gender nonconforming nor discussed sexual fantasies with them.

With these caveats in mind, childhood-onset gender dysphoria is supported to the extent that a gender dysphoric person provides consistent and persuasive evidence of extreme gender nonconformity during childhood. In these cases, childhood gender dysphoria is often but not always recalled. Autogynephilic gender dysphoria is indicated if a natal male admits being sexually aroused by cross-dressing or by the idea of having the body of a woman. ROGD is indicated for a natal female if childhood-onset gender dysphoria is absent, a rapid adolescent or young adult onset is evident. (The application of ROGD to natal males is more problematic, due to the possibility that males with apparently rapid onset have autogynephilia.) Additionally, evidence of social influences (e.g., experience with peers or social media advocating transgender identification) is more consistent with ROGD than either of the other two types.

### The Present Study

The present research explores the retrospective experiences of an Internet-recruited sample of formerly trans-identifying young adults. The following domains were assessed: motivations for the decision to adopt transgender identity; the course of mental health, psychological well-being, and gender dysphoria before, during, and after transgender identification; experiences with medical and social transition; and motivations for relinquishing a transgender identity. We also included measures intended to illuminate the extent to which our detransitioners and desisters can be understood as having had childhood-onset, autogynephilic, or rapid-onset gender dysphoria.

## Method

### Participants

Using social media, Internet sites, and word of mouth, we recruited persons ages 18–33 who had previously identified as transgender for a duration of least six months, stopped identifying as transgender, and had not identified as transgender for at least six months. Participants were surveyed about their experiences before, during, and after transgender identification.

During the recruitment period, 78 individuals who met inclusion criteria completed online surveys. The following inclusion criteria were used: 18–33 years of age; residing in the USA; previous identification as transgender for at least 6 months; lack of current identification as transgender, with cessation of transgender identification at least six months prior to participation. “Transgender” was defined as including all gender identification that is not consistent with one’s natal sex (including nonbinary, agender, enby, transgender, etc.). Ninety individuals were screened for eligibility with videoconference interviews, and five were ineligible. Three exclusions were due to transgender disidentification being too recent, one individual was not within the eligible age range, and another individual still identified as transgender. Eighty-five eligible individuals were provided with personalized one-time-use links to the online survey with assigned study identification numbers embedded into the surveys. The large majority (91.2%) of eligible individuals who received these links submitted responses.

### Procedure

Recruitment information was shared by email and social media with requests that individuals share the information with any person or community where there may be eligible individuals. Efforts were made to reach communities with differing perspectives about gender dysphoria, desistance, transition, and detransition. We contacted various organizations, individuals, and forums including: Pique Resilience Project, subreddits r/detrans and r/actual detrans, multiple individuals who have detransitioned, several individuals who are transgender, psychologists, psychiatrists, and therapists who work with gender dysphoric individuals and/or detransitioned individuals (including professionals who have worked at gender identity-affirming clinics), professional listservs for researchers and clinicians, the LGBT centers of two large universities, journalists, and more. Recruitment was open from 3/5/20 to 8/19/20 for a total of 5.5 months. The purpose of the study was described in the recruitment information, and participation was voluntary. Electronic consent was obtained before participants could view the survey questions. Data were collected through the Qualtrics Survey Platform without IP addresses.

The study was initially launched as an anonymous online survey that included screening questions that ended the survey if participants provided answers that were inconsistent with eligibility. Shortly after recruitment began, individuals began posting tweets to invite other people to take the survey with the goal of creating invalid results. This was followed by multiple tweets of individuals boasting that they submitted fake responses to the survey. In response to the sabotage attempts, the study was modified to increase the security by adding a videoconference screening interview and the use of personalized one-time-use links to the survey. The current study includes only participants who completed videoconference screening interviews.

### Measures

A survey instrument including 114 questions was created with the input of 11 professionals (including both researchers and clinicians) and 3 detransitioners. In our description of the survey instrument below, we have prioritized general information about the content domains. More specific information about some measures is provided in the Results.

#### Demographics

Participants answered demographic questions about their age, natal sex, race/ethnicity, educational attainment, political beliefs, and religiosity.

#### Development and Onset

Participants recalled the ages when they began to identify as transgender and when this identification stopped. Duration of trans-identification was computed using these ages.

Eight items adapted from DSM-5 criteria (American Psychiatric Association, 2013) asked participants to recall symptoms of gender dysphoria experienced from age 3 through 11 years. Coefficient alpha for the composite scale was 0.81.

Participants were provided with a definition of rapid-onset gender dysphoria (Littman, 2018) and were asked whether the term fit their own experience. This item appeared as, “The term ‘rapid-onset gender dysphoria’ has been used to describe a situation where someone who did not have gender dysphoria during their childhood, appears to suddenly develop gender dysphoria during or after puberty. Does this description fit your experience?” Participants responded either “Yes,” “Don’t know,” or “No.” For some analyses reported herein, this response was made numeric (with “Don’t know” considered intermediate between “Yes” and “No”).

Several items inquired about experiences, thoughts, or feelings that happened over the course of three months prior to becoming gender dysphoric or trans-identified.

Several items asked about participants’ sociopolitical attitudes. These included questions about the attitudes of participants’ families, participants’ current attitudes in general, and participants’ attitudes about gay, lesbian, and transgender rights.

#### Sexuality

##### Sexual Orientation

Sexual attraction to males versus females was assessed using the 7-point Kinsey scale with responses ranging from “exclusively sexually attracted to males” to “exclusively sexually attracted to females” (Kinsey et al., 1948). Numerically for the Kinsey scale, 0 represents exclusive other-sex attraction, 3 represents identical attraction to both sexes, 6 represents exclusive same-sex attraction, and 1, 2, 4, and 5 represent intermediate degrees of relative attraction to males and females. An additional option assessed absence of attraction to either cisgender males versus cisgender females. Participants were asked to rate their sexual attraction at three time points; before they identified as transgender, while they were identifying as transgender, and after they stopped identifying as transgender. In this paper, we restrict analyses to their most recent self-reported sexual orientation.

##### Autogynephilia/Autoandrophilia

Three items intended to measure autogynephilia for natal males (Blanchard, 1989) or autoandrophilia for natal females were included. (Currently, autoandrophilia is neither well researched nor well supported.) Two items were the same for both natal sexes: “Did you ever experience sexual arousal by dressing as the other sex in private?” and “Did you ever experience sexual arousal when fantasizing that you had the body of the other sex?” The third item differed appropriately for natal males and females: “Did you ever feel sexually aroused by the idea of being a woman? [for natal males]” and “Did you ever feel sexually aroused by the idea of being a man? [for natal females].” Items were scored dichotomously and summed so that scores ranged from 0 to 3. Cronbach’s alpha for the autogynephilia and autoandrophilia scales was 0.58 and 0.76, respectively.

#### Mental Health

##### Psychiatric Diagnoses

Participants were asked to indicate which of 13 psychiatric diagnoses they were given over the course of their lifetime and which of these psychiatric diagnoses they received before they started to identify as transgender. Psychiatric diagnoses listed included anxiety, attention deficit hyperactivity disorder (ADHD), autism spectrum disorder, bipolar disorder, borderline personality disorder, depression, eating disorders, history of pulling out hair, obsessive compulsive disorder (OCD), post-traumatic stress disorder (PTSD), schizophrenia or psychosis, selective mutism, Tourette’s, and “other.” The diagnoses were chosen because we expected that some (e.g., “anxiety” and “depression”) were especially likely to be elevated among gender dysphoric persons, and others (e.g., “schizophrenia or psychosis”) were otherwise important to assess. Some of these diagnoses, as listed (e.g., “schizophrenia or psychosis” and “anxiety”), did not strictly correspond with DSM-5 diagnoses (American Psychiatric Association, 2013).

##### Gender Dysphoria

Participants answered six items reflecting DSM-5 criteria for gender dysphoria (American Psychiatric Association, 2013), both for the period they identified as transgender and for the period after they stopped identifying as transgender. Cronbach’s alphas for these scales were 0.69 and 0.77, respectively. Additionally, a single item assessed recalled severity of gender dysphoria symptoms on an eight-point scale from, “0” (participants didn’t notice or barely noticed any distress) to “7” (participants’ stress was so severe that it strongly interfered with their ability to function in their daily life). Participants rated this item for three time periods: before identifying as transgender, during the period of transgender identification, and after transgender identification ceased. The gender dysphoria-related items were not intended to provide a formal diagnosis, and two requirements were not assessed: whether symptom duration had lasted for at least six months and whether individuals were distressed or impaired by their symptoms. Because of these omissions, our estimates for gender dysphoria diagnostic status represent upper bounds (i.e., the maximum number of participants who could have met the criteria).

##### Self-Harm

Participants indicated whether they had engaged in self-harm (e.g., cutting, burning, or picking) for three periods: before, while, and after identifying as transgender.

##### Flourishing

The Secure Flourishing Measure (VanderWeele, 2017) was used to assess participant recalled general well-being at two points in time: while transgender identified, and after transgender identification. This measure consists of 12 questions answered on a scale of 0–10. Higher scores indicate higher levels of well-being. Cronbach’s alphas for the two time periods were 0.86 and 0.84, respectively.

#### Possible Psychosocial Influences

Several kinds of psychosocial experiences have been identified as potential causes of gender dysphoria, including the misinterpretation of psychological distress as gender dysphoria (Littman, 2018, 2021). These include negative life experiences during childhood and adolescence, peer influence, and Internet-related preoccupation.

##### Recalled Childhood and Adolescent Negative Experiences

Recalled childhood and adolescent trauma was assessed using ten items from the Adverse Childhood Experiences (ACE) scale (Felitti et al., 1998). These items are answered dichotomously and concern a variety of negative life events potentially experienced in the family (primarily due to parental mistreatment) before age 18 years. Items were summed to create a composite score, and Cronbach’s alpha for this scale was 0.73.

Recalled negative experiences prior to transgender identification were also assessed using 9 items (e.g., “Before you started to identify as transgender, did you experience bullying?”). With one exception (“Witnessing the abuse of a family member (including sibling, parent, cousin, etc.”)), these items did not focus on within-family maltreatment. Items were summed to create a composite score, and Cronbach’s alpha for this scale was 0.71.

##### Friendship Group Dynamics

Several items asked about potential friendship group dynamics potentially relevant to the onset of transgender identification. For example, one item asked: “At the time you started to identify as transgender, did you belong to an online friend group or community where one or more friends became transgender-identified around the same time?”.

##### Internet Usage

The Problematic and Risky Internet Use Screening Scale (PRIUSS) (Jelenchick et al., 2014) is a 23-item scale that assesses excessive and emotionally unhealthy Internet usage. The scale was used to retrospectively assess problematic Internet usage for two time periods: during the first six months of transgender identification and the six months prior to the survey. This scale does not focus on the content of Internet preoccupations (e.g., transgender-related), only on problematic Internet behavior per se. Composites were formed by summing all items for the relevant time. Coefficient alpha for the earlier time was 0.95, and it was 0.93 for the more recent time.

##### Participants’ Ratings of Psychosocial Influences

Participants were asked to rate the importance of 39 potential psychosocial influences on their transgender identification on a scale from 1 (not at all important) to 5 (extremely important). We consider these questions individually in the Results.

#### Transition Experiences

Participants indicated which steps they had taken toward social and medical transition. Furthermore, participants who had used cross-sex hormones provided information about where they obtained them and their experiences of the informed consent process.

#### Detransition and Desistance

Participants indicated whether they felt most “authentic” before, during, or after transgender identification (or in more than one of those periods). They also rated their likelihood of future transgender identification on a 5-point scale from “Extremely likely” to “Not at all likely.”

## Results

After exclusions, participants included 78 individuals: 71 natal females and 7 natal males. Although we were keenly interested in possible differences between natal females and males, the small number of males meant that statistical power to test for such differences was very low. Thus, below we indicate only when such tests were statistically significant.

Regarding where they learned of the study, 45% (*N* = 35) of participants indicated the r/Detrans subreddit, 32% (*N* = 25) some other social media source, 10% (*N* = 8) the Pique Resilience Project, 8% (*N* = 6) from an acquaintance, and 5% (*N* = 4) indicated some other, unnamed, source.

Participants’ current age ranged from 18 to 33 years, (*M* = 24.89, SD = 4.33). The large majority of those who indicated their ethnicity identified as “white” (*N* = 63, 81%); 10% (*N* = 8) identified as multi-ethnic, 6% (*N* = 5) as “Asian,” and 3% (*N* = 2) as “Hispanic.” Regarding education, 36% (*N* = 28) had acquired a college or graduate degree, 5% (*N* = 4) an associate degree, 45% (*N* = 35) had attended college without earning a degree, and 13% (*N* = 10) had obtained a high school degree or equivalent. Only one individual had not graduated from high school.

We examined participants’ past and present general sociopolitical attitudes. In general, these tended to be liberal. For example, 70% (*N* = 40) of those who responded indicated that their childhood family environment was moderately or very liberal, compared with 23% (*N* = 16) who described their family as moderately or very conservative. A similar pattern emerged in their descriptions of their own, adult politics, with 68% (*N* = 52) describing themselves as moderately or very liberal, compared with 13% (*N* = 10) as moderately or very conservative (and these were all moderate). Consistent with social liberalism, most participants indicated that religion was not very important, with 82% (*N* = 64) agreeing that it was not at all or slightly important, compared with only 18% (*N* = 14) who agreed that it was at least moderately important.

Attitudes toward gay and transgender rights are especially pertinent, and participants’ attitudes about these were especially liberal: 86% (*N* = 67) strongly supported gay marriage rights, and 91% (*N* = 71) supported transgender rights. Only one person expressed opposition to either of these, opposing transgender rights.

### Detransition and Desistance Status

The survey defined “detransition” as stopping the usage of cross-sex hormones and/or having surgery to reverse previous gender transition. (This is a narrow and stringent sense of “detransition” because it does not include cessation of social transition.) Most participants (68%, *N* = 53) had taken at least one medical step toward transition and thus may be considered “detransitioners.” Of this group, 23% (*N* = 18) had undergone both some hormonal treatment and surgical intervention, 40% (*N* = 31) had only undergone hormonal treatment, and 5% (*N* = 4) had only had surgery. (We provide more detail about specific treatments below.) The minority of participants (32%, *N* = 25) who had not received either hormonal or surgical interventions may be considered “desisters.” All participants had taken at least one social transition step, and 83% had taken three or more.

### Development and Onset

#### Gender Nonconformity

Participants completed a questionnaire regarding childhood gender nonconformity and dysphoria (with items corresponding to diagnostic criteria for DSM-5 Gender Dysphoria in Childhood), assessed for ages 3–11 years. Table 1 provides the endorsement frequencies of the eight items, in descending order**.** In general, the most frequently endorsed items assessed gender nonconformity: behaving as the other sex and rejection of sex-typical behavior. The least commonly endorsed items focused on gender dysphoria, dislike of one’s body and desire to be the other sex.

**Table 1:** Diagnostic criteria endorsed for DSM-5 Gender Dysphoria in Childhood

Item	*N* (%) participants endorsed
Strong preference for sex-atypical toys	44 (56.4)
Strong rejection of sex typicality (i.e., masculinity for boys, femininity for girls)	39 (50.0)
Desire to dress as other sex and/or resistance to dressing as natal sex	35 (44.9)
Strong preference for cross-sex roles in play	32 (41.0)
Strong preference for playmates of other sex	25 (32.1)
Strong desire for other sex’s physical attributes	23 (29.5)
Strong dislike of sexual anatomy	21 (26.9)
Strong desire to be other sex	20 (25.6)

**Fig. 1:**
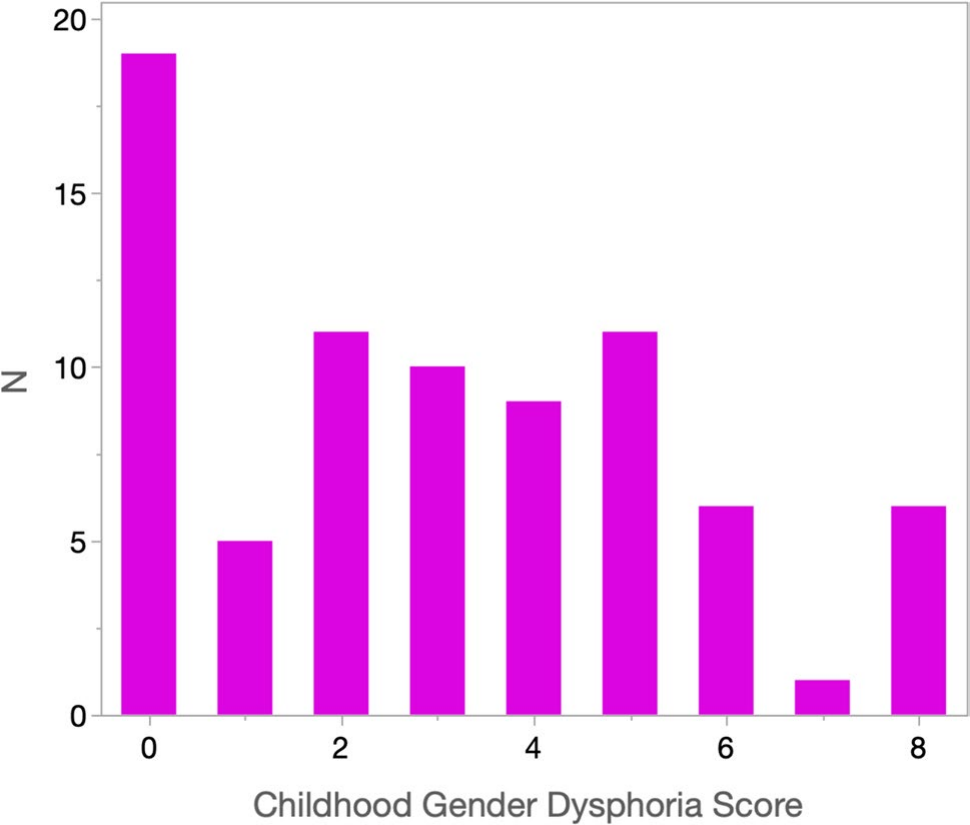
Frequency distribution of summed scores of DSM-5 diagnostic criteria for gender dysphoria in childhood

Figure 1 presents the frequency distribution of summed scores across the eight items. The most common score (24.4%, *N* = 19) was 0, indicating endorsement of none of the items. Only 7.7% (*N* = 6) obtained the highest possible score, 8. The remainder of the sample was spread evenly across the scale, with points of rarity at 1 (only one item endorsed) and 7 (all but one item endorsed). Because we did not ask about two diagnostic requirements (duration of at least six months and distress or impairment), at most 16.7% (*N* = 13) of participants could have met diagnosis of DSM-5 Gender Dysphoria in Childhood (endorsement of at least six of eight items).

#### Rapid Onset

One survey item explained the term “rapid-onset gender dysphoria” (ROGD) and asked participants whether they believed it applied to them. Fifty-three percent (*N* = 41) of the sample answered “yes,” 23% (*N* = 18) did not know, and 24% (*N* = 19) answered “no.” After transforming this into a three-point numeric scale (from 0 = “No” to 2 = “Yes”), we examined its correlation with the self-reported childhood gender dysphoria scale. This correlation, *r*(78) = −0.57, *p* < 0.0001, indicated that those who reported greater childhood gender dysphoria were much less likely than those who reported less childhood gender dysphoria to believe that rapid-onset gender dysphoria applied to them.

Participants were asked whether, while becoming gender dysphoric or transgender-identified, any of five potential changes happened over the course of three months or less: (1) adopting the belief that “gender dysphoria” was the only explanation for preexisting feelings and emotions; (2) reinterpreting past feelings and behaviors to be consistent with gender dysphoria or transgender identity; (3) labeling of feelings and experiences as “gender dysphoria” or “transgender;” (4) considering past and current feelings and experiences as proof of being transgender; and (5) acquiring the belief that transition would be the solution to one’s problems. On average, participants endorsed 4.22 (SD = 1.23) of the five items. Furthermore, the number of items endorsed was positively related to the numeric scale of whether respondents thought that rapid-onset gender dysphoria applied to them, *r*(78) = 0.23, *p* = 0.044.

Table 2 provides data on timing of when participants both started and stopped identifying as transgender. Perhaps surprisingly, age of first transgender identification was only weakly related to degree of childhood gender dysphoria, *r*(78) = −0.17, *p* = 0.15—although the direction of the association was in the intuitive direction, with greater childhood gender dysphoria predicting earlier transgender identification. Nor was participant’s degree of agreement that their gender dysphoria was “rapid-onset” significantly associated with age at trans-identification, *r*(78) = 0.01, *p* = 0.96. The length of time during which participants were transgender-identified was significantly related to the degree to which they identified with “rapid-onset,” *r*(78) = −0.24, *p* = 0.03, with endorsement of “rapid-onset” associated with shorter duration of transgender identification. Duration of transgender identification was also positively related to childhood gender dysphoria, *r*(78) = 0.25, *p* = 0.03.

**Table 2:** Time course of trans-identification and desistance

	Mean (SD)	Range
Age first identified as transgender (years)	17.12 (3.82)	6–28
Length of time identified as transgender (years)	5.35 (3.31)	1–14
Age stopped identifying as transgender (years)	22.46 (4.21)	15–32
Length of time since identifying as transgender (years)	2.42 (2.24)	0.5–12

### Sexual Orientation

#### Attraction to Males versus Females

**Fig. 2:**
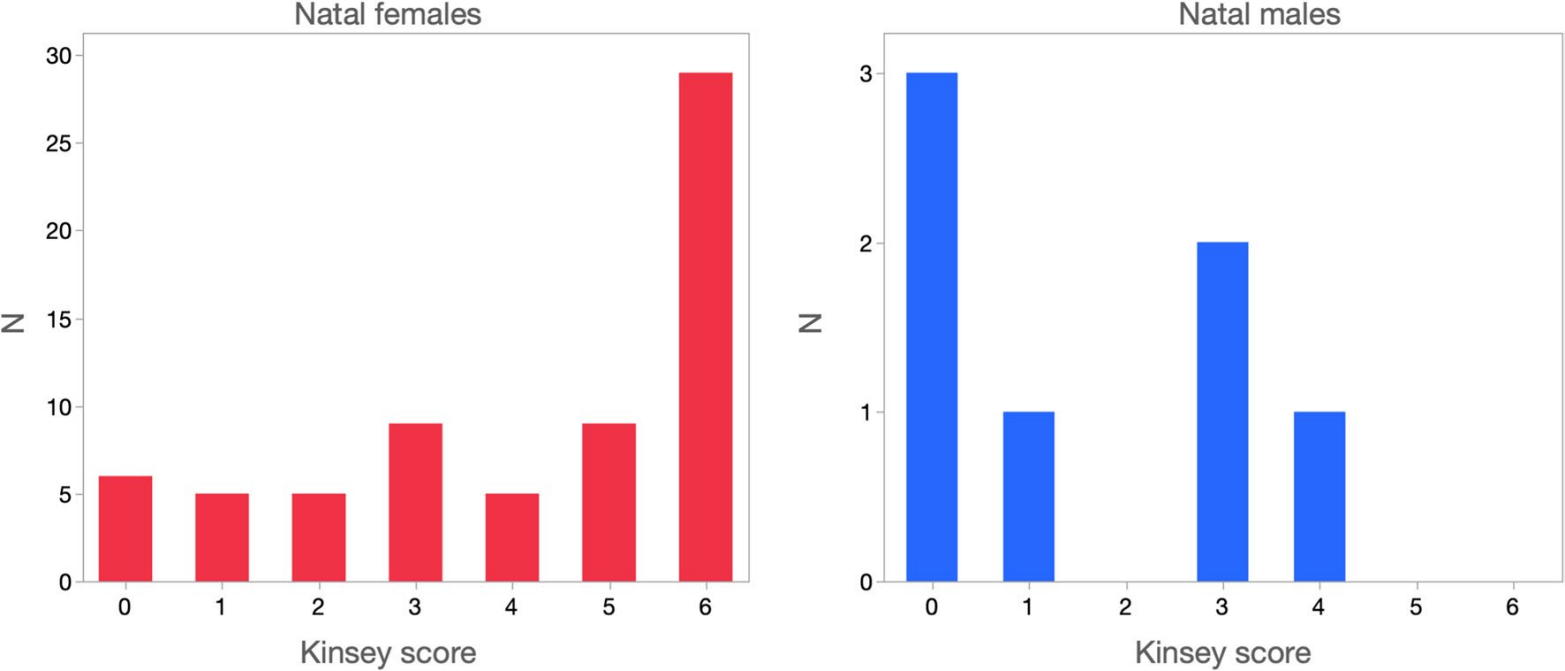
Frequency distributions of Kinsey scores for natal females and natal males

Figure 2 presents the frequency distributions of current Kinsey scores, separately for male and female participants. Kinsey scores of 0 represent exclusive attraction to the other sex, and scores of 6 exclusive attraction to one’s own sex; scores of 1–5 represent intermediate degrees of preference. Natal females’ attraction patterns were strongly female-biased, with 43 participants indicating greater attraction to women than to men, and 16 indicating greater attraction to men. The most common Kinsey score among natal females was 6, indicating exclusive attraction to women. This score was endorsed by 43% (*N* = 29) of female respondents who answered this question.

Attraction patterns of natal males were also female-biased, with 3 of 7 participants indicating exclusive attraction to women. Importantly, no natal male endorsed exclusive or near-exclusive attraction to men (i.e., Kinsey scores of 6 or 5, respectively), suggesting that none of these participants would be considered homosexual by Blanchard’s taxonomy.

In samples not recruited for being gender dysphoric, there is typically a correlation between recalled gender dysphoria/nonconformity and adult sexual orientation (Bailey & Zucker, 1995). In the current sample, the correlation between recalled childhood gender dysphoria and adult sexual orientation (i.e., Kinsey score) was *r*(68) = −0.06, *p* = 0.60 for females and *r*(7) = 0.89, *p* = 0.007 for males. In both cases, higher recalled gender dysphoria was associated with greater male attraction, although this correlation was statistically significant only for males.

#### Autogynephilia and Autoandrophilia

On average, males agreed with at least two of the three autogynephilia items, and females one of the three autoandrophilia items, *M*_M_ = 2.29 (SD = 1.25), *M*_F_ = 1.06 (SD = 1.16), *d* = 1.02, *p* = 0.009. Only one item differed significantly: 6/7 males had experienced sexual arousal while cross-dressing, compared with 15/71 females, *χ*^2^(1, *N* = 78) = 13.51, *p* = 0.0002. Among natal females, there was a substantial negative correlation between autoandrophilia and Kinsey score, *r*(68) = −0.39,* p* = 0.0009, indicating that higher autoandrophilia scores were especially common among respondents more attracted to males.

### Mental Health Before, During, and After Transgender Identification

#### Psychiatric Diagnoses

Table 3 presents the frequencies that participants said they had received the 13 psychiatric diagnoses before their transgender identification. The table also includes the lifetime frequencies of these diagnoses. (Lifetime diagnoses include all prior diagnoses.) The rate of any diagnosis was quite high, with only 5% (*N* = 4) of participants having none of the 13 diagnoses queried during their lifetime. The mean numbers of diagnoses from this list reported by participants were 2.46 (*MD* = 2; *SD* = 1.97) before transgender identification, and 3.65 (*MD* = 4; *SD* = 1.98) lifetime. The most common diagnoses, both before transgender identification and during the lifetime, were anxiety (60.26% before transgender identification and 79.94% lifetime) and depression (62.82% before transgender identification and 79.49% lifetime).

**Table 3:** Frequency of psychiatric diagnoses before trans-identification and during lifetime

Diagnosis	Before trans-identification *N* (%)	Lifetime *N* (%)
Anxiety	47 (60.26%)	62 (79.49%)
ADHD	19 (24.36%)	32 (41.03%)
Autism spectrum disorder	7 (8.97%)	17 (21.79%)
Bipolar	9 (11.54%)	17 (21.79%)
Borderline personality disorder	3 (3.85%)	8 (10.26%)
Depression	49 (62.82%)	62 (79.49%)
Eating disorder	18 (23.08%)	23 (29.49%)
Hair pulling	8 (10.26%)	8 (10.26%)
OCD	14 (17.95%)	15 (19.23%)
PTSD	12 (15.38%)	30 (38.46%)
Schizophrenia	4 (5.13%)	9 (11.54%)
Selective mutism	1 (1.28%)	2 (2.56%)
None of the above	7 (8.97%)	4 (5.13%)
Other	4 (5.13%)	11 (14.10%)

##### Self-Harm

**Fig. 3:**
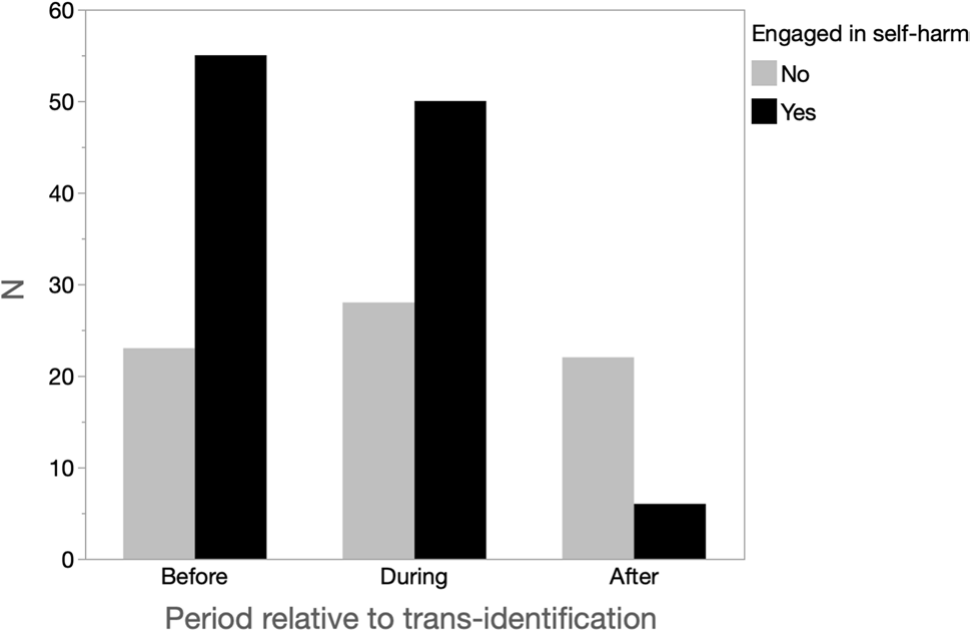
Frequency distributions of self-harm for all participants for the periods before, during, and after trans-identification

Participants also indicated whether they had engaged in self-harm during each of the three periods (before, during, and after transgender identification), and the frequency distributions for all participants are presented in Fig. 3. The lifetime rate of self-harm was high, 79% (*N* = 62). Natal females were more likely to have any history of self-harm (83%; *N* = 59) compared with natal males (43%; *N* = 3), Fisher’s exact test = 0.03. Compared with both earlier periods, the period after cessation of transgender identification was associated with markedly less self-harm, with 71%, 64%, and 23% of participants saying they had self-harmed before, during, and after transgender identification. Treating the dichotomous variable of harm as numeric (which is defensible for proportions that are not extreme; see Hellevik, 2009), the contrast between self-harm after transgender identification was significantly lower than the average of the other two periods, paired *t*(77) = 8.85, *p* < 0.0001, which did not differ, paired *t*(77) = 1.09, *p* = 0.28.

##### Gender Dysphoria

**Fig. 4:**
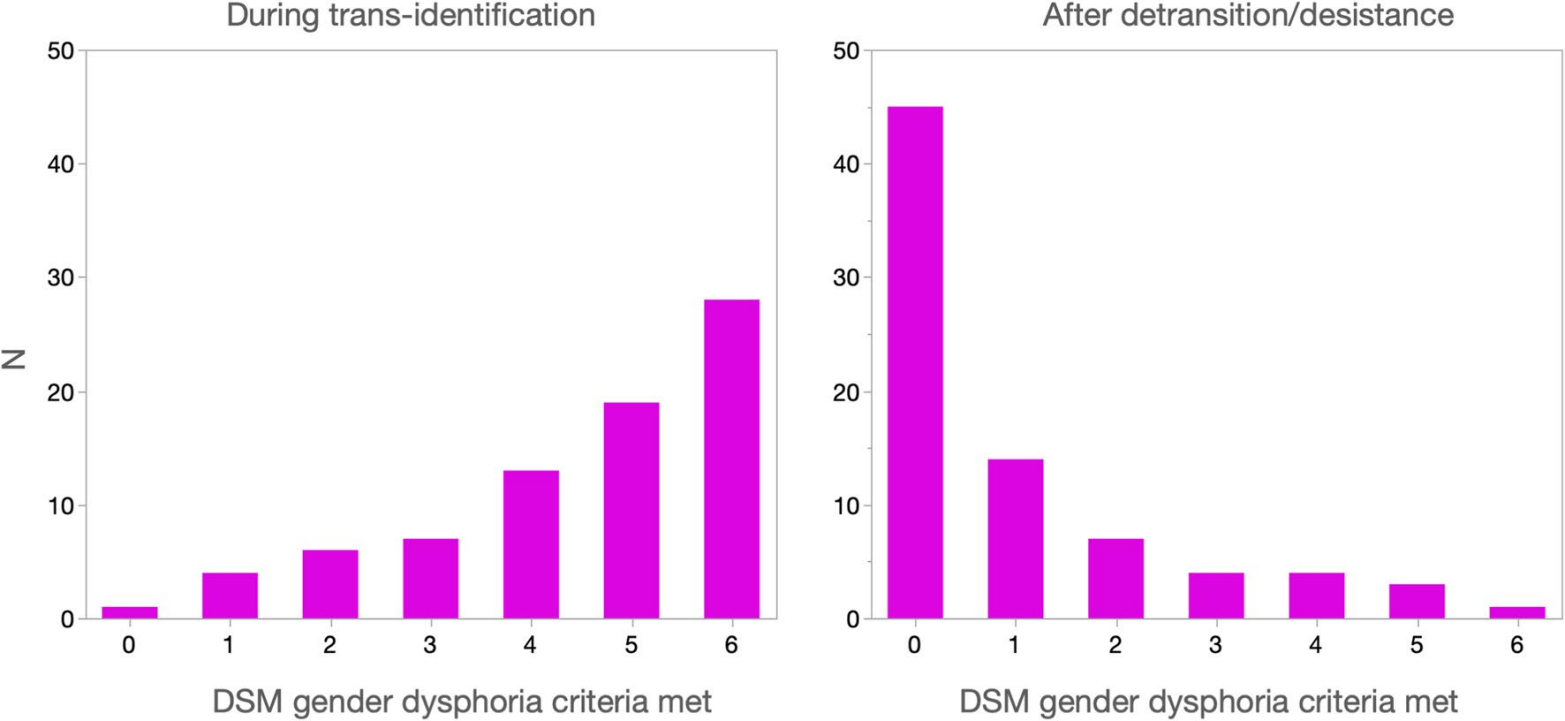
Frequency distributions of the number of DSM-5 gender dysphoria participants met during trans-identification and after detransition/desistance

We assessed gender dysphoria across time in two ways. In the first, we asked participants whether they agreed with six statements derived from DSM-5 criteria for Gender Dysphoria in Adolescents and Adults (e.g., “Did you feel a strong desire to be the opposite natal sex?”). These questions were asked both for the period during trans-identification and for the period since trans-identification ended. The frequency distribution for this variable is presented for these two periods in Fig. 4. There was a marked decrease in gender dysphoria from trans-identification, *M* = 4.51 (SD = 1.59) to after trans-identification, *M* = 0.98 (SD = 1.52), *d* = 2.27, paired *t*(77) = 16.65, *p* < 0.0001.

**Fig. 5:**
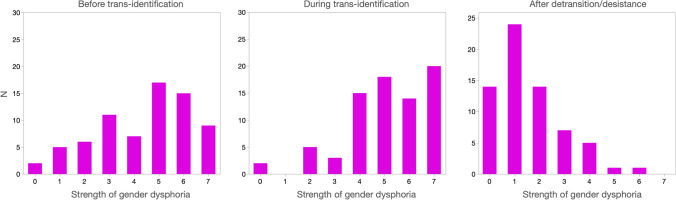
Frequency distributions of participants’ self-rated strength of gender dysphoria before, during, and after trans-identification

Strength of gender dysphoria was also assessed using a single item with responses ranging from 0 (no distress over natal sex) to 7 (distress severe enough to interfere with ability to function in daily life). Figure 5 shows frequency distributions for responses to this item for three periods: before trans-identification, during trans-identification, and after trans-identification. Dysphoria rose considerably in the sample after participants began identifying as trans and dropped drastically after trans-identification ceased. To analyze these trends properly, we conducted the following within-subjects analyses: Two orthogonal polynomial variables were constructed (Judd et al., 2017). The first, linear contrast, representing the decline in dysphoria from before trans-identification to after trans-identification, was 2.8 points, *t*(60) = 10.7, *p* < 0.0001. The second, quadratic contrast, comparing dysphoria during trans-identification to the average of the periods before and after trans-identification, was 2.3 points, *t*(59) = 14.4, *p* < 0.0001. Thus, participants’ trans-identification phase was especially dysphoric, and their post-trans-identification phase especially non-dysphoric.

##### Flourishing

**Fig. 6:**
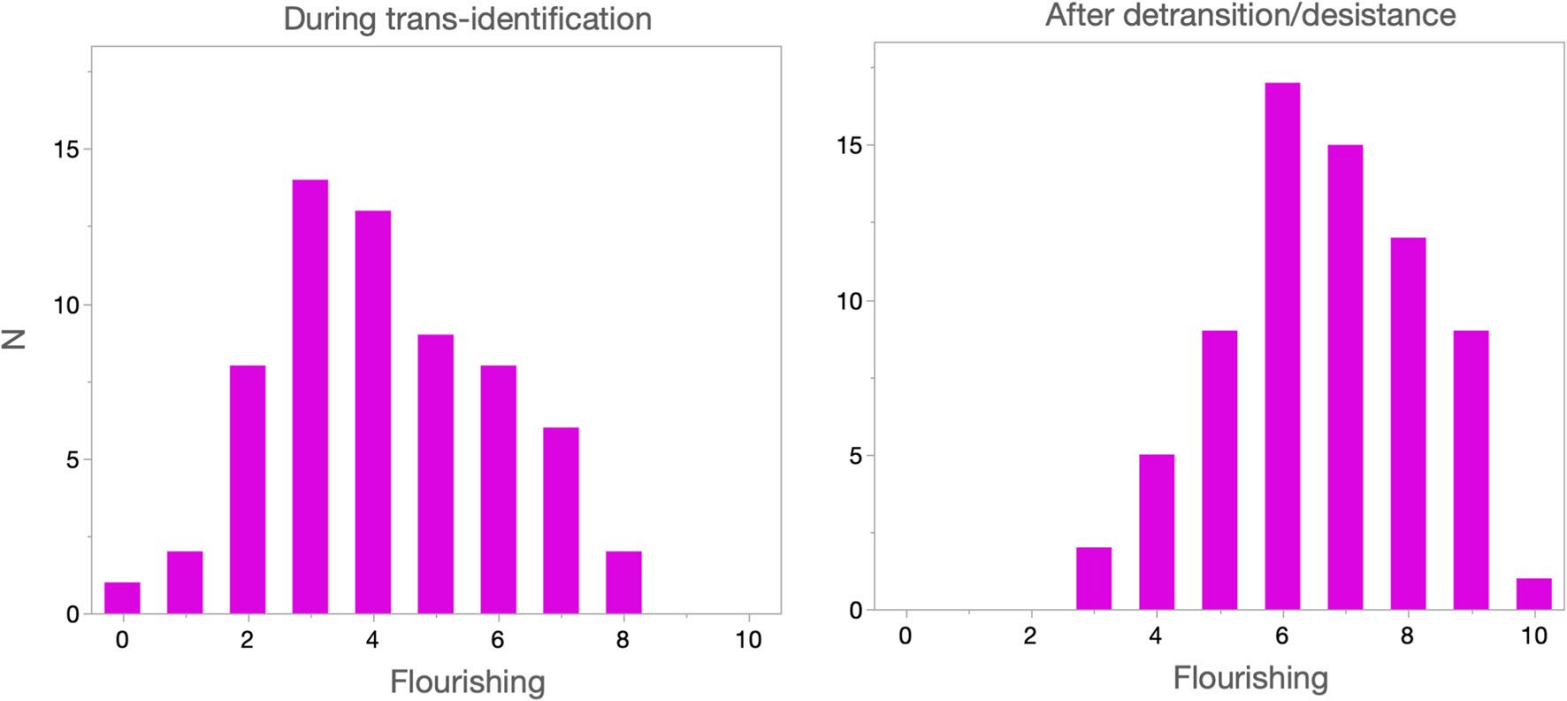
Frequency distribution of Flourishing for the periods during trans-identification and after detransition/desistance

Participants completed the Secure Flourishing Measure to assess general well-being, for two time periods: while they identified as transgender and after they stopped identifying as transgender. Figure 6 shows the frequency distributions of self-reported Flourishing during and after participants’ trans-identification. On average, participants reported that after transgender identification ended Flourishing increased by 2.55 points on a 10-point scale, *d* = 1.49, paired *t*(57) = 9.26, *p* < 0.0001.

#### Possible Psychosocial Influences on Transgender Identification and Gender Dysphoria

Participants were asked to rate the importance of 39 potential psychosocial influences on their becoming trans-identified on a scale from “not at all important” (which we assigned a value of 1) to “extremely important” (5). Mean ratings are presented in Table 4 in descending order of magnitude. The item most closely related to conventional understanding of gender dysphoria, “Being born in the wrong body,” obtained a mean rating of 2.89 (SD = 1.44), substantially lower than the highest-rated item, “Interpreting the feelings of trauma or a mental health condition as gender dysphoria” (*M* = 3.96, SD = 1.33) and also lower than 22 other potential influences. Endorsement of “Being born in the wrong body” was negatively correlated with the self-endorsement of rapid-onset gender dysphoria, *r*(74) = −0.37, *p* = 0.001. Even participants who did not believe that ROGD applied to them rated “Interpreting the feelings of trauma or a mental health condition as gender dysphoria” as slightly more relevant than “Being in the wrong body” to their transgender identification (ratings of 3.7 versus 3.6, respectively), although that difference was not statistically significant.

**Table 4:** Ratings of importance of potential psychosocial influences on becoming transgender-identified and gender dysphoric

Potential influence	Mean Importance (SD)
Interpreting the feelings of trauma or a mental health condition as gender dysphoria	3.96 (1.33)
Internal feelings of misogyny (or misandry)	3.87 (1.31)
Wanting to avoid how women (or men) are treated in society	3.83 (1.38)
Exposure to other people’s misogyny (or misandry)	3.78 (1.37)
Self-hatred and wanting to be a completely different person	3.78 (1.49)
Wanting to avoid sexual expectations or oversexualization	3.77 (1.39)
Not fitting in with members of their natal sex	3.69 (1.21)
Maladaptive coping mechanism	3.69 (1.42)
Needing to figure out one’s identity	3.65 (1.16)
Trying to cope and avoid painful feelings	3.61 (1.37)
Identifying with opposite-sex characters in books, movies, video games, etc.	3.51 (1.50)
Believing that they were not good enough in the roles and behaviors expected of their natal sex	3.37 (1.48)
Tumblr	3.34 (1.42)
Believing that one was not feminine enough (if female) or masculine enough (if male)	3.25 (1.44)
Not being interested in the things that most other members of natal sex were interested in	3.23 (1.36)
Social influence	3.18 (1.43)
It was an important part of identity development at the time	3.18 (1.30)
Wanting to avoid feeling vulnerable to sexual predators	3.16 (1.70)
A person known offline (in real life)	3.16 (1.53)
Sexual trauma	3.15 (1.65)
YouTube transition videos	3.10 (1.58)
Sexual harassment	2.97 (1.53)
Being born in the wrong body	2.89 (1.44)
Love of or fascination with masculinity (if female) or femininity (if male)	2.88 (1.41)
Difficulty accepting self as lesbian (if female), gay (if male), or bisexual	2.86(1.65)
Social contagion	2.84 (1.51)
A community of people met online	2.83 (1.33)
A person met online	2.79 (1.49)
YouTube transgender celebrities	2.78 (1.58)
A group of people known offline (in real life)	2.70 (1.68)
Desire to belong to a friend group	2.66 (1.46)
A dating, romantic or sexual partner	2.66 (1.64)
Perceptions of self and society that are related to being a person with Aspergers	2.64 (1.76)
Perceptions of self and society that are related to being a person with autism	2.64 (1.85)
Wanting to avoid the homophobia that would be experienced for being lesbian (if female), gay (if male) or bisexual	2.55 (1.58)
Being bullied	2.48 (1.36)
Sexual excitement when fantasizing about being the other sex	2.42 (1.59)
Experiencing homophobic bullying	2.29 (1.25)
Negative reaction to pornography	2.28 (1.45)
Falling in love or liking (romantically) someone who is not attracted to people of one’s own natal sex	2.24 (1.59)
Peer pressure	2.18 (1.48)
A school-based club or organization (like a GSA or University LGBT advocacy club)	2.15 (1.45)
Reddit	2.14 (1.47)
Wanting to be part of a social movement	2.06 (1.34)
A therapist	2.03 (1.42)
The desire to remain in an existing friend group	1.96 (1.44)
Positive reaction to pornography (liking or being influenced by pornography)	1.86 (1.30)
Cosplay community	1.83 (1.40)
Exposure to high levels of hormones prenatally	1.81 (1.15)
Not wanting to be part of the “oppressor group”	1.80 (1.22)
Thinking that their parents would be homophobic toward them	1.71 (1.23)
A speaker who gave a presentation at school	1.51 (1.19)
A Group therapy setting	1.48 (1.15)
DeviantArt	1.44 (0.88)
A family member	1.29 (0.71)
A religious community	1.20 (0.76)
A gaming community	1.04 (0.21)
Community or friends at a summer camp	1.02 (0.15)

Mean importance score derived from scale 1 = “not at all important;” 2 = “somewhat important;” 3 = “moderately important;” 4 = “very important;” 5 = “extremely important.” Responses of “N/A” were excluded. N/A responses were those that did not apply to a participant and so could not be rated for importance

##### Trauma

A common belief among clinicians who favor ROGD theory is that traumatic events can contribute to the occurrence of gender dysphoria (Evans & Evans, 2021; Withers, 2020). Participants completed the Adverse Childhood Experiences (ACE) checklist, a 10-item scale assessing the experiences of 10 putative traumatic factors prior to age 18. These items pertained to experiences within the home family. The mean score was 3.94 (SD = 2.34), which is relatively high. For example, in a large representative study conducted by the CDC, only 15.2% of women and 9.2% of men had scores as high as 4, the median and approximate mean of the current sample.

Participants also indicated whether they had experienced any of nine negative experiences likely to have been experienced more recently—although prior to transgender identification—and not necessarily within the home. Table 5 provides the frequencies for these items. The mean number of these experiences reported by participants was 3.60 (SD = 2.20).

**Table 5:** Additional negative experiences recalled prior to trans-identification

Traumatic experience	Number reporting experience	Percentage reporting experience
Peer exclusion	61	78.2
Bullying	50	64.1
Sexual harassment	44	56.4
Homophobic bullying	36	46.2
Sexual abuse	27	34.6
Witnessing abuse of a family member	24	30.8
Abuse by dating partner	17	21.8
Rape	15	19.2
Attempted rape	7	9.0

The correlation between the self-reported number of adverse childhood experiences and the number of more recent negative experiences was high, *r*(77) = 0.59. Table 6 presents correlations for these two negative experiences scales with several potentially relevant variables. ACE scores were significantly associated with duration of trans-identification (higher ACE scores predicting longer duration), belief that “rapid-onset gender dysphoria” applies to oneself (higher ACE scores predicting less agreement), and number of mental disorders before trans-identification (higher ACE scores predicting more disorders).

**Table 6:** Correlations with recalled negative experiences

Variable	Correlation with childhood adverse experiences		Correlation with later negative experiences	
	*r*	*p*	*r*	*p*
Gender Dysphoria before trans-identification	0.08	0.50	0.07	0.58
Age at start of trans-identification	– 0.09	0.41	0.10	0.39
Duration of trans-identification	0.41	< 0.001	0.25	0.03
Agreement that ROGD applies to self	– 0.23	0.04	0.04	0.72
Number of mental disorders before trans-identification	0.23	0.047	0.35	0.002
Number of mental disorders, lifetime	0.14	0.21	0.39	< 0.001

##### Peer Influences

Previous work identified unique friendship group dynamics associated with the onset of transgender identification. These included friendship groups mocking people who were not transgender-identified or LGBTIA and friendship groups where more than 50% of the friendship group became transgender-identified (Littman, 2018). Participants in the current study were asked if, at the time of transgender identification, they belonged to a friendship group where one or more members of the group became transgender-identified around the same time. The majority (60.3%) answered in the affirmative (with 24.4% referring to offline friendship groups, 14.1% referring to online friendship groups, and 21.8% referring to both). More than a third of participants responded that the majority of their offline and online friends became transgender-identified (34.6% and 38.5%, respectively) and participants acknowledged that their offline and online friendship groups engaged in mocking people who were not transgender-identified (42.3% and 41.0%, respectively).

Hypotheses regarding social contagion of gender dysphoria have emphasized the idea that trans-identification often follows immersion in certain Internet sites with intense discussion of transgender phenomena, such as Tumblr (Littman, 2018). Participants completed the PRIUSS for two time periods. Participants’ scores for the six-month period after they started to identify as transgender *M* = 34.03 (SD = 24.03) were substantially higher than those for the six-month period prior to the survey, *M* = 19.34 (SD = 14.72), *d* = 0.83, paired *t*(76) = 7.86, *p* < 0.0001. Furthermore, the average of earlier scores was substantially higher than the recommended cutoff for “problematic Internet usage,” and the average of later scores was substantially lower than that cutoff. Participants’ scores correlated substantially across the two time periods, *r*(77) = 0.61, despite the large drop in average scores, suggesting persistent individual differences in Internet usage. The correlation between participants’ earlier PRIUSS score and the degree to which they endorsed the idea that rapid-onset gender dysphoria applied to themselves was low, *r*(77) = −0.03, *p* = 0.77, counter to predictions.

#### Transition Experiences

##### Social and Medical Transition Steps

We asked participants about social and medical steps they had taken during their transition. Table 7 presents these steps, separated by natal sex where appropriate. On average, participants had taken 3.62 of the social steps (SD = 1.05), and all had taken at least one. Most participants had used a different name, different pronouns, and had modified their appearance (clothes, hair, makeup). Most natal females had used a binder to give the impression of a flat chest. Nearly half had used a prosthetic penis (i.e., packer). Among natal males, the use of prosthetic breasts or female genitals (i.e., gaffs) were comparatively rare. The mean number of social steps taken by natal females, 3.61 (SD = 1.02), was greater than this number for natal males, 2.86 (SD = 1.07), *t*(76) = 2.05, *p* = 0.043. Regarding medical transition steps, all natal males and most natal females had used cross-sex hormones (estrogen and testosterone, respectively). Almost a third of natal females had undergone breast removal, a small number had their uterus or ovaries removed, and none had received phalloplasty. No natal males had undergone gender-affirming surgeries.

**Table 7:** Steps taken for social and medical transition

	*N* (%)
Social transition	
Different name	68 (87.18%)
Pronouns	71 (91.03%)
Clothes/Hair/Makeup	73 (93.59%)
Natal female	
Binder	63 (88.73%)
Prosthetic penis	30 (42.25%)
Natal male	
Breast form	1 (16.67%)
Gaff	1 (16.67%)
Medical transition	
Puberty blockers	2 (2.56%)
Hormones	49 (62.82%)
Surgery	22 (28.21%)
Natal female	
Testosterone	42 (59.15%)
Breast removal	21 (29.58%)
Uterus removal	3 (4.23%)
Ovaries removal	2 (2.82%)
Natal male	
Estrogen	7 (100.00%)
Anti-androgen	6 (85.71%)
Breast augmentation	0 (0.00%)
Testes removal	0 (0.00%)
Penis removal	0 (0.00%)
Vaginoplasty	0 (0.00%)

More than half (66.7%, *N* = 52) of the participants sought medical care to obtain cross-sex hormones and the majority of those seeking cross-sex hormones (92.3%, *N* = 48) received them. Four participants sought but did not receive cross-sex hormones. Reasons included parental refusal (*N* = 3); participant decision not to obtain cross-sex hormones (*N* = 2) and refusal of the clinician to prescribe cross-sex hormones (*N* = 1).

Only 27.1% of participants informed the clinician or clinic that facilitated their transition that they had detransitioned.

##### Informed Consent

Asked who provided cross-sex hormones, participants had most often consulted primary care physicians (41%), followed by psychiatrists who treat adults (19%), endocrinologists (18%), psychiatrists who treat children and adolescents (10%), social workers (12%), and nurse practitioners (8%). About 40% of participants had obtained cross-sex hormones at a clinic specializing in gender issues, with the remainder going to a general health clinic (27%), a private practice (14%), planned parenthood (5%), a private gender clinic (1%), or other sources. Participants who started cross-sex hormones continued to take them for a mean duration of 2.59 years (SD = 2.03).

Most (61.5%, *N* = 32) participants had obtained cross-sex hormones from clinical practices using the “informed consent” model of care. The other participants indicated either that the practice did not use informed consent (23.1%, *N* = 12) or that they were uncertain whether it did so (15.4%, *N* = 8). With respect to the adequacy of informed consent, most participants were informed about both risks (89.6%) and benefits (77.1%) of cross-sex hormones. However, many believed that the information provided was not adequate: 66.7% felt they were inadequately informed about risks and 31.3% felt this about benefits. Only one participant (2.1%) reported that a clinician provided information about treatment alternatives to cross-sex hormones (including the possibility of not taking cross-sex hormones), and 75.0% of participants reported that they received inadequate information about these alternatives.

Participants were asked whether they were informed about scientific evidence regarding late-onset gender dysphoria. Fewer than one-tenth (8.3%) of participants indicated that they were informed by their clinician about the lack of long-term studies about natal females with late-onset gender dysphoria. Similarly, only 12.5% were informed that the risks, benefits, and outcomes for medical transition of late-onset gender dysphoric youth have not been well studied.

#### Desistance and Detransition

Participants were asked to rate the importance of 20 factors on the cessation of their transgender identification using a scale from “not at all important” (coded for analyses as 1) to “extremely important” (5). Mean ratings are reported in Table 8 in descending order of magnitude. The factors with the highest rating of importance were the participant’s “own thought processes” (*M* = 4.74, SD = 0.65); “feeling that the causes for [their] gender dysphoria were more complicated than [they] previously understood them to be” (*M* = 4.25, SD = 1.22); and the participant’s “personal definition of ‘female’ and ‘male’ changed and [they] now felt comfortable identifying as natal sex” (*M* = 4.03, SD = 1.42). Factors that might be described as external pressures to desist or detransition obtained the lowest ratings of importance scores, including “transphobia or discrimination while transgender identified” (*M* = 1.46, SD = 0.84); “pressure from family” (*M* = 1.37, SD = 0.75); “religion or religious beliefs” (*M* = 1.15, SD = 0.61); and “peer pressure” (*M* = 1.11, SD = 0.48).

**Table 8:** Ratings of importance of various factors to the cessation of transgender identification

	*N*	Mean (SD)
Participant’s own thought processes	78	4.74 (0.65)
Feeling that the causes of gender dysphoria were more complicated than participant previously understood	75	4.25 (1.22)
Understanding of “female” and “male” changed so that participant now felt comfortable identifying as natal sex	75	4.03 (1.42)
Feeling that “transgender” no longer fit participant	73	3.86 (1.37)
Discovering a specific cause of gender dysphoria, such as trauma or a mental health condition	71	3.68 (1.45)
Feeling uncomfortable with the transgender community	74	3.45 (1.50)
Lack of improvement in mental health while identifying as transgender	76	3.41 (1.46)
Change in participant’s political or philosophical views	71	3.25 (1.52)
Unmet expectations about life improvement	73	3.04 (1.52)
Worsened mental health while identifying as transgender	76	3.03 (1.62)
Resolution of strong emotions that led to transgender identification	65	2.72 (1.43)
Transgender identification no longer served a purpose	76	2.67 (1.33)
Dissatisfaction with physical changes from transition	59	2.59 (1.49)
Wishing to return to cisgender	69	2.46 (1.47)
Missing life from before coming out or transition	70	2.19 (1.38)
Difficulty finding someone for a dating, romantic, or sexual relationship	62	1.79 (1.38)
Transphobia or discrimination while transgender identified	72	1.46 (0.84)
Pressure from family	63	1.37 (0.75)
Religion or religious beliefs	52	1.15 (0.61)
Peer pressure	63	1.11 (0.48)

Mean importance score derived from a scale where 1 = “not at all important”; 2 = ”somewhat important”; 3 = ”moderately important”; 4 = ”very important”; 5 = ”extremely important.” “Not applicable” responses were excluded from counts, and so N represents the number of responses with numeric ratings

Anecdotally, it is common for transgender individuals to report feeling most “authentic” following gender transition. We asked participants if they felt most authentic before identifying as transgender, while identifying as transgender, or after they no longer identified as transgender. (Participants could select more than one option). The overwhelming majority of participants (95%) reported feeling most authentic after detransition/desistance. Only 9% felt most authentic while identifying as transgender.

We asked participants to rate the likelihood that they might re-identify transgender in the future. Only three participants viewed this outcome as likely (one as “very likely” and two as “moderately likely”). The remaining participants indicated that this was somewhat unlikely (20.5%) or not at all likely (75.6%).

## Discussion

Results of our exploratory and wide-ranging study of detransition and desistance among previously transgender-identified young adults are necessarily tentative. Our results suggest that the following applies to many of our participants: Adolescents and young adults struggling with mental health issues began to experience gender dysphoria—often suddenly and without prior history of gender issues. Subsequently these individuals identified as transgender. Transgender identification was not fleeting, but typically lasted for several years, and was associated with serious social and medical steps. All our informants took steps to socially transition, and most also obtained and used cross-sex hormones. An appreciable minority also had “gender-affirming” surgery. During transgender identification, gender dysphoria and general unhappiness increased considerably.

In our study, the factors most important to relinquishing a transgender identification were internal factors, such as participants own thought processes, changes in participants’ personal definitions of male and female, and becoming more comfortable identifying as their natal sex. External factors such as discrimination and pressure from family were rated as least important. The greater importance of internal factors than external factors is consistent with the findings from other studies of detransitioners (Littman, 2021; Vandenbussche, 2022) and differs from results of studies of currently transgender-identifying individuals (James et al., 2016; Turban et al., 2021). After detransition and desistance, informants became much happier and much less gender dysphoric. They reported little inclination to regret detransition and desistance. Before elaborating these and other findings, we consider our study’s scientific limitations.

### Limitations

A methodologically near-ideal study of detransition and desistance would follow a randomly selected group of transgender-identified youth over time, assessing relevant factors (e.g., gender dysphoria, transition steps, current adjustment, and sexuality) repeatedly. Furthermore, to reduce distortions due to self-report bias, additional informants (e.g., parents and therapists) would be enlisted. This design would allow the estimation of the likelihood that transgender-identified individuals would take various transition steps, that their well-being would improve, and that they would detransition or desist—among other important potential findings. Furthermore, this design would allow exploration of which factors predict important later outcomes.

Our study deviated from the near-ideal design in several respects. Our sample of detransitioners and desisters was recruited by distributing announcements through social media and relevant Internet sites. Hence, we cannot know whether our informants were representative of detransitioners and desisters. Nor can we know how they differed from transgender-identifying individuals who have not detransitioned or desisted. Our study relied exclusively on detransitioners’ and desisters’ self-reports. Furthermore, informants were surveyed only once, but they reported on their own feelings and behavior across a wide range of time, from childhood through early adulthood.

Although our study’s limitations seriously constrain our ability to answer some questions with certainty, they constrain us less in some other important domains. Obviously, our design does not allow us to estimate how common detransition and desistance are. Nor can we know which if any variables predict detransition and desistance. Some variables that we studied, including childhood gender dysphoria, negative life events, and current sexual orientation, may sometimes be inaccurately reported. Conclusions depending on these data are especially tentative. However, participants’ experiences of gender dysphoria and of flourishing before, during, and after transgender identification are more likely to be accurately remembered. Other information provided by respondents that we see little reason to question includes psychiatric diagnoses, Internet usage, social and medical transition steps, experiences obtaining cross-sex hormones, and experiences with informed consent.

A final reason why our study makes a valuable contribution is that little is currently known about detransitioners and desisters, especially during the recent past. Indeed, little is known about any aspect of gender dysphoria that begins after childhood, especially among natal females. This is true even though the incidence of adolescent-onset gender dysphoria among natal females has been briskly growing (Zucker, 2019). When little is known, imperfect research is often better than no research. It can provide provisional answers, better-informed hypotheses, and ideas for future research. In the remaining Discussion, we attempt to provide these.

### Who Are the Detransitioned?

Only a history of adopting and then relinquishing a transgender identity—the primary inclusion criteria for our study—was true of all participants. Some other, less uniform, trends were also evident. For example, participants were far more likely to be natal females than natal males. Some other kinds of gender dysphoria occur more often among natal males than among natal females (Bailey & Blanchard, 2017; Zucker et al., 2012). Despite the small number of natal male participants in our study, results suggested some important sex differences. Nearly all the natal male participants reported a history of sexual arousal while cross-dressing, a primary sign of autogynephilia. Autogynephilic gender dysphoria is one of two well-established types of gender dysphoria in natal males. The other type, homosexual gender dysphoria, occurs among natal males whose gender nonconformity (and usually gender dysphoria) is obvious during childhood and whose sexual attraction is exclusively toward other males. None of our male participants reported exclusive attraction to other males. Our results are consistent with the possibility that all the natal male participants were autogynephilic. Neither autogynephilia nor its gender-reversed version, autoandrophilia, has been established as an important cause of gender dysphoria among natal females.

Our participants reported seemingly high levels of previous mental problems. These usually predated transgender identification, with more than 90% of participants reporting a prior clinical diagnosis. Mean (and median) number of lifetime diagnoses exceeded 2. Unfortunately, we are not aware of good comparison estimates for representative samples of youth for the number of lifetime diagnoses. A large and epidemiologically representative 2005 study estimated that by age 75 about half of US adults will be diagnosed with a mental disorder (Kessler et al., 2005). The rates self-reported in our study were already much higher than this, even though our participants were much younger than 75 years.

The sample also reported a high lifetime rate of self-harm: 83%. Unfortunately, again, we are unaware of a close comparison sample. A US cohort of adults younger than 30 years produced a rate of 19%. A more recent study of British adults found a lifetime rate of self-harm of 5% (Liu, 2023). A recent study of Norwegian university students found a lifetime rate of self-harm of 19.6%, much higher than the British sample but much lower than the present one.

One variable that did not allow generalization but instead suggested considerable divergence, is self-reported childhood gender dysphoria. Scores on the relevant measure were widely dispersed. The most common score was zero (i.e., no childhood gender dysphoria), but a considerable minority (30.8%, 24/78) of scores on that variable exceeded the middle of the scale. Validity of this variable is especially problematic because it relies on childhood memory, Furthermore, exaggerated memories of childhood gender nonconformity and dysphoria may be encouraged in both clinical and peer contexts (Littman, 2018). Thus, our results concerning childhood gender nonconformity are especially tentative. Future research would benefit by including reports of childhood behavior using additional informants (e.g., parents).

### Causes of Gender Dysphoria

Keeping in mind these caveats, what do our results suggest about causes of gender dysphoria in our sample? Approximately one-half of the sample endorsed the applicability of “rapid-onset gender dysphoria” (ROGD) to themselves, one-quarter was uncertain, and one-quarter disagreed with this application. This is consistent with the finding that most participants did not recall high levels of childhood gender dysphoria. Furthermore, those reporting lower degrees of childhood gender dysphoria were more likely to endorse an explanation of ROGD for themselves.

Two other aspects hypothesized to contribute to ROGD were surveyed: emotional turmoil unrelated to gender dysphoria, and social influence (Littman, 2018). Participants rated adjustment to mental health challenges and to trauma as more important causes than the feeling of “being born in the wrong body” as reasons for transgender identification (Table 4), although those who did not believe that ROGD applied to them rated them similarly. Regarding social influences, a substantial minority of participants reported previous immersion in peer groups with high levels of transgender identification. Furthermore, participants reported a high level of problematic Internet usage during the first six months of transgender identification.

We have noted that participants reported high levels of stress and trauma, and some believed these experiences were important in the development of their gender dysphoria. However, drawing causal conclusions about the role of trauma in causing psychological problems has been difficult in general. This is because there are often multiple possible explanations for associations between trauma and psychological problems (Bailey & Shriver, 1999). This difficult scientific issue cannot be resolved in this study.

Finally, the concept of rapid-onset gender dysphoria may be more valid for natal females than for natal males. Autogynephilic natal males may appear to have a rapid onset, but they are typically aware of autogynephilic arousal since puberty, when strong sexual feelings begin. To be sure, both autogynephilic males and ROGD females may, in principle, be socially influenced toward adopting a transgender identity. However, their underlying motivations differ.

### Transition Experiences

Our participants had undergone substantial gender transition. On average, participants identified as transgender for nearly five years (Md = 4 years). During this time, all participants took at least one step toward socially transition, such as name and pronoun changes, as well as changing their physical presentation; most participants took several steps. Most obtained cross-sex hormones, and a substantial minority also underwent serious surgeries: 30% of female participants had had their breasts removed. Although most participants recalled receiving information about the risks and benefits of cross-sex hormones, a majority did not feel the information they were provided about risks was adequate.

### Detransition and Desistance

Detransition and desistance were associated with marked improvements in psychological functioning. On several relevant measures—gender dysphoria, flourishing, and self-harm—participants indicated great improvement after they stopped identifying as transgender. These findings depend on retrospective self-report, but this seems appropriate.

Our study cannot resolve whether detransition and desistance caused these changes in our participants. It is possible, for example, that improvement in psychological functioning preceded detransition, or that detransition and improvement were both caused by a third factor. Participants believed that their detransition reflected realizations that they had mistaken ideas about gender dysphoria, lack of improvement during trans-identification, and changes in their self-conceptualizations (Table 8). They rejected family and peer pressure, transphobia, and religious beliefs as explanations of detransition.

One issue that we cannot resolve in this study is whether our participants are unique in respects that made them poor candidates for transition. Perhaps many or most youth who have transitioned at similar ages—in our sample the mean was approximately 17 years—adjust well to their gender change. Our participants invested a great deal of their lives in their gender transitions—in terms of time, disruption, and serious social and medical steps. Thus, we do not believe that a principled case can be made that participants detransitioned because they were never gender dysphoric.

### Future Directions

Follow-up studies of gender dysphoric youth are urgently needed. Ideally, gender dysphoric youth should be recruited using a variety of sources, including social media, treatment facilities, gender clinicians, and parent groups. When possible, information should be obtained from multiple sources, especially youth and their parents. Results of both the present study and prior research support the desirability of collecting data on several important variables: childhood gender nonconformity and dysphoria, sexuality, psychiatric diagnoses, parental attitudes toward transition, transgender prevalence in peer groups, current gender dysphoria, and current psychological adjustment. Organizations providing clinical services to gender dysphoric youth have a particular obligation to follow these youth and assess their outcomes. Unfortunately, in North America at least, we see little evidence that this presently occurs.

### Conclusions

We surveyed a sample of young adults who previously identified as transgender but had detransitioned or desisted. Most participants were born female. Mental health issues, including prior diagnoses and a history of self-harm, were especially common. A history of gender dysphoria during childhood was reported by a nontrivial minority of participants. A slight majority believed their histories were consistent with rapid-onset gender dysphoria. Factors most associated with detransition were internal factors, reflecting psychological change, rather than external factors, such as family or social pressure. Detransitioned participants reported that they had become much less gender dysphoric, and much happier, than they were during their period of trans-identification.

### Supplementary Information

Below is the link to the electronic supplementary material.

Supplementary file1 (PDF 457 KB)
