# Supplementary material for: Detransition and Desistance Among Previously Trans-Identified Young Adults
Source: Arch Sex Behav. 2023 Dec 1;53(1):57–76. doi: 10.1007/s10508-023-02716-1 (PMC10794437; doi:10.1007/s10508-023-02716-1)
Supplement: Supplementary file 1 — Supplementary file1 (PDF 457 KB) [file 10508_2023_2716_MOESM1_ESM.pdf]

D&D Launch 2

Survey Flow

EmbeddedData
Study IDValue will be set from Panel or URL.

Standard: Consent (2 Questions)
Standard: Welcome page (1 Question)
Block: Research Survey: Demographics and Background (19 Questions)
Standard: Research Survey: Before Transgender-identification (10 Questions)
Standard: Research Survey: Becoming Transgender-identified (21 Questions)
Standard: Research Survey: While Transgender-identified (23 Questions)
Standard: Research Survey: Informed Consent for medical transition (44 Questions)
Standard: Research Survey: Desisting and After Desistance (24 Questions)

Page Break

Start of Block: Consent

You are invited to take part in a Brown University research study. Your participation is voluntary. RESEARCHER: Lisa Littman, MD, MPH PURPOSE: The purpose of this research is to learn about the experiences of desisters and detransitioners--specifically to explore: 1) which factors may or may not be related to the development of and desistance from transgender identification; 2) whether or not individuals experienced changes in their sexual orientation during and after transgender-identification;and 3) what kinds of counseling and informed consent were received by those who sought medical care to transition. You are being asked to be in this study because you are currently 18 to 33 years old, live in the U.S. and: previously identified as transgender for at least six months; do not currently identify as transgender; and have not identified as transgender for at least six months.

PROCEDURES: You will be asked to complete a confidential web-based survey.

TIME INVOLVED: The survey will take 60-90 minutes of your time.

COMPENSATION: You will not receive compensation for your time. RISKS: The possible risks to you in taking part in this research include feeling uncomfortable answering questions or potential loss of confidentiality. You may skip any questions that you do not wish to answer.

This survey will ask some questions about trauma, self-harm, and mental health. **These assessments will not be reviewed immediately**. Everyone participating in this research study will receive a list of contacts if they want to speak with someone about any health concerns or abuse. Please use the following list of contacts and resources if needed: The National Alliance on Mental Illness (NAMI): https://www.nami.org/Find-Support; helpline: 1-800-950-6264; and Crisis textline: 741741 The National Sexual Assault Hotline: https://www.rainn.org/about-national-sexual-assault-telephone-hotline; helpline: 1-800-656-4673 The National Suicide Prevention Lifeline: https://suicidepreventionlifeline.org; helpline: 1-800-273-8255 Substance Abuse and Mental Health Services Association: https://www.samhsa.gov/find-help/national-helpline; helpline: 1-800-662-4357

To protect your identity as a research subject, your personal identifying information (your name, year of birth, email address) will be kept separate from your research data (the answers you provide in the survey). Dr. Littman, and researchers who work directly with her, will be able to link your data to you by using a study ID number -but we will destroy this link and all of your personal identifying information no later than one week after recruitment is complete The survey will not ask for personal information that could identify you and will not collect IP addresses.

There will be open-text questions in the survey where you can type your answers in your own words. Quotes may be taken from these open-text answers and used in publication and presentations but without specific identifying details. If personal identifiers are written, they will not be included in the publications or presentations to protect your privacy. Additionally, if a quote contains potentially recognizable information, the detail will be omitted or adjusted to be made more anonymous. BENEFITS: You may not directly benefit from being in this

research study CONFIDENTIALITY: To protect your privacy, data (survey responses) will be collected by Qualtrics which is a secure system. Additionally, your personal identifying information (name, year of birth, email address) and the data collected from the survey will be stored separately in the Qualtrics system. Your personal identifying information and the linking document will be destroyed no later than one week after study recruitment is complete. The responses to the surveys will be anonymized.The anonymized data may be used and/or shared for future research. VOLUNTARY: You do not have to be in this study if you do not want to be. Even if you decide to be in this study, you can change your mind. You can withdraw from the study at any point before the linking information is destroyed. You can also stop answering survey questions at any point. If you answer some questions and then stop, the responses to questions you did answer will be saved as research data, unless you withdraw from the study. CONTACT INFORMATION: If you have any questions about your participation in this study, you can contact Lisa Littman, MD, MPH at Lisa_Littman@brown.edu. YOUR RIGHTS: If you have questions about your rights as a research participant, you can contact Brown University's Human Research Protection Program at 401-863-3050 or email them at IRB@Brown.edu. CONSENT TO PARTICIPATE: Clicking the "agree" below confirms that you have read and understood the information in this document, are 18-33 years of age, and that you agree to volunteer as a research participant for this study. You can print a copy of this form at: https://www.littmanresearch.com/new-research

---

I have read and understand the information in this document, I am 18-33 years of age, and I voluntarily agree to participate.

○ Agree (1)

○ Disagree (2)

Skip To: End of Survey If I have read and understand the information in this document, I am 18-33 years of age, and I volun... = Disagree

End of Block: Consent

Start of Block: Welcome page

Thank you for participating in this study. There is very little known about people who desist or detransition from a transgender identification. The information that you provide here is incredibly important. The survey is long, so please leave yourself enough time to complete it. This should take about 60-90 minutes. If you take a break from the survey and come back to complete it, the responses you already entered should be saved as long as you use the same computer and browser.

Several questions that appear to repeat are asked for different periods of your life so please read the questions carefully. To make it easier to keep track, questions are grouped by time-period, so there is a section of questions pertaining to before you identified as transgender, another section pertaining to while you identified as transgender, etc.

The language and terminology around sex and gender are rapidly changing and several different terms are in use at the same time. The following is a list of terms and how they will be used in this survey.

Gender identity refers to the way an individual perceives themself in terms of masculinity and femininity (examples: man, women, transwomen, transman, non binary, agender)

Natal sex and **sex at birth** will be used to refer to a person’s sex that was observed at the time of birth and, if genetic screening or sonogram was performed, prior to birth (Examples: natal female, natal male).

Gender Dysphoria refers to a persistent discomfort from the difference between a person’s experienced gender identity and their natal sex

Transgender is often used as an umbrella term to describe many gender identities (such as transgender, non-binary, enby, agender, genderfluid, genderqueer, etc) where a person’s gender identity is different than their natal sex. In the survey questions, transgender will be used as a broad term that encompasses all gender identities that differ from natal sex.

Transgender-identified refers to a person identifying as (feeling that they are) transgender (or any gender identity that differ from a person’s natal sex)

Desistance is when someone’s symptoms of gender dysphoria resolve or go away or when someone who previously identified as transgender no longer identifies as transgender. (A desister is a person who has desisted.)

Detransition is when a person who has transitioned by using cross-sex hormones and/or surgery, stops the process and either ceases use of cross-sex hormones and/or has surgery to reverse the changes that occurred from the gender transition. (A detransitioner is a person who has detransitioned.)

End of Block: Welcome page

Start of Block: Research Survey: Demographics and Background

What is your age?

▼ 18 years old (2) ... 33 years old (18)

What is your natal sex (sex at birth, sex)?

- ○ Female (1)
- ○ Male (2)
- ○ Other (3)

What age were you when you started to identify as transgender?

▼ 1 year old (2) ... 33 years old (34)

How long did you identify as transgender?

____

What age were you when you stopped identifying as transgender?

▼ 1 year old (1) ... 33 years old (34)

How long has it been since you last identified as transgender?

____

What is your race/ethnicity (check all that apply)?

- ☐ Asian (1)
- ☐ Black or African American (4)
- ☐ Hispanic or Latina/Latino (5)
- ☐ Native American or Alaska Native (6)
- ☐ Native Hawaiian or Pacific Islander (7)
- ☐ White (8)
- ☐ Other (9)

What is the highest level of school you have completed?

- ○ Less than a high school degree (1)
- ○ High school or equivalent such as GED (4)
- ○ Some college but no degree (5)
- ○ Associate degree (6)
- ○ Bachelor’s degree (7)
- ○ Graduate degree, such as (MA, PhD, MD, JD, etc) (8)

---

1. How would you describe your socioeconomic status, financial situation or wealth relative to other people in your country (for the time you were a child and currently)?

	Very much above average for my country (8)	Somewhat above average for my country (9)	About average for my country (10)	Somewhat below average for my country (11)	Much lower than the average for my country (12)	I prefer not to say (13)
During childhood (1)	○	○	○	○	○	○
Currently (4)	○	○	○	○	○	○

Is your medical insurance…?

○ Private (4)

○ Public (such as Medicaid) (5)

○ Other (6)

How would you describe your political beliefs (during your childhood and currently)?

	Very liberal (1)	Moderately liberal (2)	Moderate (4)	Moderately conservative (5)	Very conservative (6)	No political beliefs (7)
During childhood (1)	○	○	○	○	○	○
Currently (10)	○	○	○	○	○	○

Currently, how important is religion in your daily life?

- Extremely important (1)
- Very important (4)
- Moderately important (5)
- Slightly important (6)
- Not at all important (7)

How often do you attend religious services?

- More than once a week (1)
- Once a week (4)
- 2-3 times a month (5)
- Once a month (about 12 times a year) (6)
- 3-11 times a year (7)
- Once or twice a year (8)
- Never (9)

Do you strongly favor, favor, oppose or strongly oppose allowing gay and lesbian couples to marry legally?

- ○ Strongly favor (1)
- ○ Favor (4)
- ○ Oppose (5)
- ○ Strongly oppose (6)
- ○ Don’t know (7)

Do you believe that transgender people deserve the same rights and protections as other people in your country?

- ○ Yes (1)
- ○ No (4)
- ○ Don’t know (5)

Where did you first hear about this study?

- ○ r/Detrans subreddit (1)
- ○ Pique Resilience Project (4)
- ○ Your doctor, therapist, or social worker (5)
- ○ Social media not listed above (6)
- ○ Person you know offline (7)
- ○ Other (8)

Has a teacher or other professional told you that you were...?

- ○ Academically gifted (1)
- ○ Learning disabled, intellectually disabled, or had learning disablities (4)
- ○ Both (5)
- ○ Neither (6)

While you were growing up, during your first 18 years of life:

	Yes (1)	No (2)
Did a parent or another adult in the household OFTEN... swear at you, insult you, put you down, or humiliate you? OR act in a way that made you afraid you might be physically hurt? (1)	○	○
Did a parent or another adult in the household OFTEN...push, grab, slap or throw something at you? OR EVER hit you so hard that you had marks or were injured? (4)	○	○
Did an adult or person at least 5 years older than you EVER...touch or fondle you or have you touch their body in a sexual way? OR try to or actually have oral, anal, or vaginal sex with you? (5)	○	○
Did you OFTEN feel that... no one in your family loved you or thought you were important or special? OR your family didn't look out for each other, feel close to each other, or support each other? (6)	○	○
Did you OFTEN feel that...you didn't have enough to eat, had to wear dirty clothes, and had no one to protect you? OR your parents were too drunk or high to take care of you or take you to the doctor if you needed it? (7)	○	○
Were your parents EVER separated or divorced? (8)	○	○

Was your mother or stepmother OFTEN pushed, grabbed, slapped, or had something thrown at her? OR SOMETIMES OR OFTEN kicked, bitten, hit with a fist, or hit with something hard? OR ever repeatedly hit over at least a few minutes or threatened with a gun or knife? (9)	○	○
Did you live with anyone who was a problem drinker or alcoholic or who used street drugs? (10)	○	○
Was a household member depressed or mentally ill or did a household member attempt suicide? (11)	○	○
Did a household member go to prison? (12)	○	○

Did you, your parents, or grandparents experience any of the following?

	You (1)	Either of your parents (2)	Any of your grandparents (3)
Been targeted for racial, ethnic, or religious genocide (1)	▢	▢	▢
Had to flee from your/their home for safety reasons (4)	▢	▢	▢
Had to flee from your/their country for safety reason (5)	▢	▢	▢
Had to permanently leave your/their home because it was destroyed by a natural disaster (such as a tsunami, earthquake, flood, etc) (6)	▢	▢	▢
Been held as a prisoner (7)	▢	▢	▢
Lived in a territory that was under military occupation (8)	▢	▢	▢
Survived a war (9)	▢	▢	▢

End of Block: Research Survey: Demographics and Background

Start of Block: Research Survey: Before Transgender-identification

Before you started to identify as transgender, had you been diagnosed with any of the following (choose all that apply)?

- ☐ Anxiety (1)
- ☐ Attention Deficit Hyperactivity Disorder (ADHD) (4)
- ☐ Autism Spectrum Disorders (5)
- ☐ Bipolar Disorder (6)
- ☐ Borderline Personality Disorder (7)
- ☐ Depression (8)
- ☐ Eating Disorders (9)
- ☐ History of pulling out hair (example eyelashes or eyebrows) when anxious (10)
- ☐ Obsessive Compulsive Disorder (11)
- ☐ Post-Traumatic Stress Disorder (PTSD) (12)
- ☐ Schizophrenia or Psychosis (13)
- ☐ Selective mutism (14)
- ☐ Tourette’s (15)
- ☐ None of the above (16)
- ☐ Other (17)

If you checked any of the conditions in the previous question, please describe when it was diagnosed and how long it persisted.

____

____

____

____

____

Before you started to identify as transgender, did you experience the following (choose all that apply)

- ▢ Exclusion from your peer group (1)
- ▢ Bullying (4)
- ▢ Witnessing the abuse of a family member (including sibling, parent, cousin, etc) (5)
- ▢ Homophobic bullying (6)
- ▢ Sexual harassment (7)
- ▢ Sexual abuse (8)
- ▢ Rape (9)
- ▢ Attempted rape (10)
- ▢ Physical, emotional, or sexual abuse from a dating partner (11)
- ▢ None of the above (12)

During your childhood, when you were between the ages of 3 years and 11 years, did you have any of the following (choose all that apply)?

☐ A strong desire to be the other (natal) sex (1)

☐ A strong preference for dressing as the other natal sex and a strong resistance to wearing clothing that is more typical for your natal sex (4)

☐ A strong preference for cross-sex roles in make believe play (5)

☐ A strong preference for toys, games or activities that are stereotypically used by the other natal sex (6)

☐ A strong preference for playmates of the other natal sex (7)

☐ A strong rejection of typically masculine (if you are natal male) or typically feminine (if you are natal female) toys, games and activities (8)

☐ A strong dislike of your sexual anatomy (9)

☐ A strong desire for the physical attributes that match the other natal sex (10)

☐ None of the above (11)

If you checked any of the items in the last question, for how long did each of these feelings last? (for example: preference of playmates -2 years; strong desire to be the other natal sex-6 months, etc),

____

____

____

____

____

Before you started to identify as transgender, how did you identify your sexual orientation (choose all that apply)?

- ☐ Asexual (1)
- ☐ Bisexual (4)
- ☐ Demisexual (5)
- ☐ Gay (6)
- ☐ Heterosexual (7)
- ☐ Lesbian (8)
- ☐ Pansexual (9)
- ☐ Queer (10)
- ☐ Other (11)

For all sexual orientations that you checked in the past question, please indicate what age you identified with each label. For example: Heterosexual (until age 12); Gay(12-15); Bisexual (15-25)

____

____

____

____

____

Before you started to identify as transgender, what was your sexual attraction to cisgender males versus cisgender females? (attraction to transgender and non-binary individuals will be asked in the next question)

- ○ Exclusively sexually attracted to males (4)
- ○ Mostly sexually attracted to males (5)
- ○ Somewhat more sexually attracted to males (6)
- ○ Equally sexually attracted to males and females (7)
- ○ Somewhat more sexually attracted to females (8)
- ○ Mostly sexually attracted to females (9)
- ○ Exclusively sexually attracted to females (10)
- ○ Not sexually attracted to males or females (11)

Before you identified as transgender, how would you describe your sexual attraction to the following (cisgender, transgender and non-binary) females and males with 0 meaning "not at all attracted" and 100 meaning "extremely attracted"?

	0 = Not at all attracted 100 = Extremely attracted<br>0 10 20 30 40 50 60 70 80 90 100
Cisgender women, cisgender girls (natal female sex) ()	
Non-binary & AFAB (natal female sex) ()	
Transmen, transboys & AFAB (natal female sex) ()	
Cisgender men, cisgender boys (natal male sex) ()	
Non-binary & AMAB (natal male sex) ()	
Transwomen, transgirls & AMAB (natal male sex) ()	

Before you identified as transgender, did you have romantic or sexual relationships with people of the following genders and sexes (choose all that apply)?

	Yes (1)	No (2)
Cisgender women, cisgender girls (natal female sex) (1)	○	○
Non-binary & AFAB (natal female sex) (4)	○	○
Transmen, transboys & AFAB (natal female sex) (5)	○	○
Cisgender men, cisgender boys (natal male sex) (6)	○	○
Non-binary & AMAB (natal male sex) (7)	○	○
Transwomen, transgirls & AMAB (natal male sex) (8)	○	○

End of Block: Research Survey: Before Transgender-identification

Start of Block: Research Survey: Becoming Transgender-identified

In your own words, do you think that anything contributed to your starting to identify as transgender? if so, what?

____

____

____

____

____

In your opinion, how important were each of the following items to your becoming transgender identified and gender dysphoric?

	N/A (1)	Not at all important (7)	Somewhat important (8)	Moderately important (9)	Very important (10)	Extremely important (11)
Needing to figure out your identity/who you were (1)	○	○	○	○	○	○
The desire to belong to a friend group (4)	○	○	○	○	○	○
The desire to remain in an existing friend group (5)	○	○	○	○	○	○
You were born in the wrong body (6)	○	○	○	○	○	○
You didn't fit in with other members of your natal sex (7)	○	○	○	○	○	○
You weren't interested in the things that most others in your natal sex were interested in (8)	○	○	○	○	○	○
Believing that you did not have the qualities to be "good enough" in the role/behaviors expected for your natal sex (9)	○	○	○	○	○	○
Sexual trauma (10)	○	○	○	○	○	○
Sexual harassment (11)	○	○	○	○	○	○
Being bullied (12)	○	○	○	○	○	○

Exposure to high levels of testosterone or estrogen prior to birth (13)	○	○	○	○	○	○
Experiencing homophobic bullying (14)	○	○	○	○	○	○
Difficulty accepting that you are a lesbian (if female), or gay (if male) or bisexual (15)	○	○	○	○	○	○
Wanting to avoid the homophobia you would receive for being lesbian (if female), or gay (if male) or bisexual (16)	○	○	○	○	○	○
Thinking that your parents would be homophobic towards you (17)	○	○	○	○	○	○
Falling in love/liking someone romantically who is not attracted to people of your natal sex (18)	○	○	○	○	○	○
Love of or fascination with femininity (if male)/Love of or fascination with masculinity (if female) (19)	○	○	○	○	○	○

Exposure to other people's misogyny (dislike or disrespect of women) or misandry (dislike or disrespect of men) (20)	○	○	○	○	○	○
Internal feelings of misogyny (dislike or disrespect of women) or misandry (dislike or disrespect of men) (21)	○	○	○	○	○	○
Identifying with or relating to opposite-sex characters in books, movies, video games, etc (22)	○	○	○	○	○	○
Not wanting to be part of the "oppressor" group (23)	○	○	○	○	○	○
Trying to cope and avoid painful feelings (24)	○	○	○	○	○	○
Interpreting the feelings of trauma or a mental health condition as gender dysphoria (25)	○	○	○	○	○	○
It was an important part of your identity development at the time (26)	○	○	○	○	○	○
Social influence (27)	○	○	○	○	○	○

Peer pressure (28)	○	○	○	○	○	○
Social contagion (29)	○	○	○	○	○	○
Maladaptive coping mechanism (30)	○	○	○	○	○	○
Wanting to avoid feeling vulnerable to sexual predators (31)	○	○	○	○	○	○
Wanting to avoid how women/men are treated by society (32)	○	○	○	○	○	○
Wanting to avoid sexual expectations or oversexualization (33)	○	○	○	○	○	○
Negative reaction to pornography (34)	○	○	○	○	○	○
Positive reaction to pornography (liking or being influenced by pornography) (35)	○	○	○	○	○	○
Believing that you weren't feminine enough (if female) or masculine enough (if male) (36)	○	○	○	○	○	○
Perceptions of yourself and society that are related to being a person with autism (37)	○	○	○	○	○	○

Perceptions of yourself and society that are related to being a person with aspergers (38)	○	○	○	○	○	○
Wanting to be part of a social movement (39)	○	○	○	○	○	○
Hating yourself and wanting to become a completely different person (40)	○	○	○	○	○	○
Sexual excitement when fantasizing about being the other sex (41)	○	○	○	○	○	○

How important were each of the following sources to your becoming transgender identified and gender dysphoric?

	N/A (1)	Not at all important (7)	Somewhat important (8)	Moderately important (9)	Very important (10)	Extremely important (11)
Tumblr (1)	○	○	○	○	○	○
Reddit (4)	○	○	○	○	○	○
Deviant Art (5)	○	○	○	○	○	○
Youtube transgender celebrities (6)	○	○	○	○	○	○
Youtube transition videos (7)	○	○	○	○	○	○
Cosplay community (8)	○	○	○	○	○	○
A person you met online (9)	○	○	○	○	○	○
A community of people you met online (10)	○	○	○	○	○	○
A person that you know offline (in real life) (11)	○	○	○	○	○	○
A group of people that you know offline (in real life) (12)	○	○	○	○	○	○
A dating, romantic or sexual partner (13)	○	○	○	○	○	○

A family member (14)	○	○	○	○	○	○
A gaming community (15)	○	○	○	○	○	○
A community or friends at a summer camp (16)	○	○	○	○	○	○
A school-based club or organization (like a GSA or university LGBT advocacy club) (17)	○	○	○	○	○	○
A religious community (18)	○	○	○	○	○	○
A speaker who gave a presentation at school (19)	○	○	○	○	○	○
A therapist (20)	○	○	○	○	○	○
A group therapy setting (21)	○	○	○	○	○	○

At the time that you started to identify as transgender, how would you rate your gender dysphoria on a scale of 0-7, with "0" meaning that you don't notice (or barely notice) any distress over your natal sex and "7" meaning that your distress over your natal sex is so severe that it strongly interferes with your ability to function in your daily life? On a scale from 0-7...

0 = Barely noticeable 7 = Severe distress

0 1 2 3 4 5 6 7

Gender dysphoria rating ()	

At the time that you started to identify as transgender, did you belong to...?

○ An offline (in real life) friend group where one or more friends became transgender-identified around the same time? (1)

○ An online friend group or community where one or more friends became transgender-identified around the same time? (4)

○ Both (5)

○ Neither (6)

Approximately how many of your friends (in real life/offline and online) became transgender-identified before you?

____

Approximately how many of your friends (in real life/offline and online) became transgender-identified after you?

____

Did the majority of your in real life/offline friend group become transgender-identified?

○ Yes (1)

○ No (2)

○ N/A (3)

Did the majority of your online friend group become transgender-identified?

- Yes (1)
- No (2)
- N/A (5)

Did your in real life/offline friend group mock, put down, or make fun of people who were not transgender-identified?

- Yes (1)
- No (4)
- N/A (5)

Did your online friend group mock, put down, or make fun of people who were not transgender-identified?

- Yes (1)
- No (2)
- N/A (3)

At the time that you started to identify as transgender, did you have an increase in your social media or online activities?

- Yes (1)
- No (4)
- N/A (5)

Please answer the following for the six-month period that you first started to identify as transgender.

	Never (1)	Rarely (6)	Sometimes (7)	Often (8)	Very often (9)
How often did you go online to look up an answer to a question you had? (1)	○	○	○	○	○
How often did you find information online that was useful and accurate? (4)	○	○	○	○	○
How often did you go online to seek support? (5)	○	○	○	○	○
How often did you get the support you were looking for online? (6)	○	○	○	○	○
How often did you make friends on the internet that you still have today? (7)	○	○	○	○	○

Please answer the following for the six-month period that you first started to identify as transgender.

	Never (1)	Rarely (6)	Sometimes (7)	Often (8)	Very often (9)
How often did you choose to socialize online instead of in-person? (1)	○	○	○	○	○
How often did you have problems with face to face communication due to your internet use? (4)	○	○	○	○	○
How often did you experience increased social anxiety due to your internet use? (5)	○	○	○	○	○
How often did you fail to make real-life relationships because of the internet? (6)	○	○	○	○	○
How often did you skip out on social events to spend time online? (7)	○	○	○	○	○
How often did your offline relationships suffer due to your internet use? (8)	○	○	○	○	○

How often did you feel irritated when you were not able to use the internet? (9)	○	○	○	○	○
How often did you feel angry because you were away from the internet? (10)	○	○	○	○	○
How often did you feel anxious because you were away from the internet? (11)	○	○	○	○	○
How often did you feel vulnerable when the internet wasn't available? (12)	○	○	○	○	○
How often did you experience feelings of withdrawal from not using the internet? (13)	○	○	○	○	○
How often did you put internet use in front of important, everyday activities? (14)	○	○	○	○	○
How often did you avoid other activities in order to stay online? (15)	○	○	○	○	○

How often did you neglect your responsibilities because of the internet? (16)	○	○	○	○	○
How often did you lose motivation to do other things that needed to get done because of the internet? (17)	○	○	○	○	○
How often did you lose sleep due to nighttime internet use? (18)	○	○	○	○	○
How often did time on the internet negatively affect your schoolwork? (19)	○	○	○	○	○
How often did you feel that you used the internet excessively? (20)	○	○	○	○	○

When you first started to see online information about transgender issues, was it…(choose all that apply) ?

- ☐ Because it started to appear on political sites/blogs that you were following (1)
- ☐ Because someone you followed online starting posting/sharing it (4)
- ☐ Because someone you know in real life starting posting/sharing it (5)
- ☐ Because you searched for it (6)
- ☐ None of the above (7)

Display This Question:

If What is your natal sex (sex at birth, sex)? = Female

Please answer the following questions about potential erotic or sexual components to transgender identification.

	Yes (1)	No (2)
Did you ever experience sexual arousal by dressing as the other sex in private? (1)	○	○
Did you fantasize often about having the body of the other sex? (4)	○	○
Did you ever experience sexual arousal when fantasizing that you had the body of the other sex? (5)	○	○
Did you ever seek out pornography featuring transwomen (natal males in the process of transitioning to women)? (6)	○	○
Did you ever seek out pornography featuring transmen (natal females in the process of transitioning to men)? (7)	○	○
Did watching any specific type of pornography (such as transgender porn or forced feminization porn) increase your desire to transition? (8)	○	○
Did you often have sexual fantasies about being a straight man having sex with a straight woman? (9)	○	○
Did you often have sexual fantasies about being a gay man having sex with another gay man? (10)	○	○
Did you ever feel sexually aroused by the idea of being a man? (11)	○	○

Display This Question:

If What is your natal sex (sex at birth, sex)? = Male

Please answer the following questions about potential erotic or sexual components to transgender identification.

	Yes (1)	No (2)
Did you ever experience sexual arousal by dressing as the other sex in private? (1)	○	○
Did you fantasize often about having the body of the other sex? (4)	○	○
Did you ever experience sexual arousal when fantasizing that you had the body of the other sex? (5)	○	○
Did you ever seek out pornography featuring transwomen (natal males in the process of transitioning to women)? (6)	○	○
Did you ever seek out pornography featuring transmen (natal females in the process of transitioning to men)? (7)	○	○
Did watching any specific type of pornography (such as transgender porn or forced feminization porn) increase your desire to transition? (8)	○	○
Before you identified as transgender, did you ever wear female lingerie such as panties or bras in private? (17)	○	○
Before you identified as transgender, did you ever experience sexual arousal when wearing female lingerie such as panties or bras in private? (13)	○	○
Did you often have sexual fantasies about being a straight woman having sex with a straight man? (14)	○	○

Did you often have sexual fantasies about being a lesbian woman having sex with another lesbian woman? (15)	○	○
Did you ever feel sexually aroused by the idea of being a woman? (16)	○	○

Display This Question:

If What is your natal sex (sex at birth, sex)? = Other

Please answer the following questions about potential erotic or sexual components to transgender identification.

	Yes (1)	No (2)
Did you ever experience sexual arousal by dressing as the other sex in private? (1)	○	○
Did you fantasize often about having the body of the other sex? (4)	○	○
Did you ever experience sexual arousal when fantasizing that you had the body of the other sex? (5)	○	○
Did you ever seek out pornography featuring transwomen (natal males in the process of transitioning to women)? (6)	○	○
Did you ever seek out pornography featuring transmen (natal females in the process of transitioning to men)? (7)	○	○
Did watching any specific type of pornography (such as transgender porn or forced feminization porn) increase your desire to transition? (8)	○	○
Did you often have sexual fantasies about being a straight man having sex with a straight woman? (9)	○	○
Did you often have sexual fantasies about being a gay man having sex with another gay man? (10)	○	○
Did you ever feel sexually aroused by the idea of being a man? (11)	○	○
Did you often have sexual fantasies about being a straight woman having sex with a straight man? (14)	○	○

Did you often have sexual fantasies about being a lesbian woman having sex with another lesbian woman? (15)	○	○
Did you ever feel sexually aroused by the idea of being a woman? (16)	○	○

Did you have high expectations that transitioning would solve your problems in the following areas (choose all that apply)?

- ☐ Mental health (1)
- ☐ Romantic relationships and dating (4)
- ☐ Friends (5)
- ☐ Academic (6)
- ☐ Occupation (7)
- ☐ None of the above (8)

In the development of your becoming gender dysphoric or transgender-identified, which of the following could be described as happening over the course of three months or less (choose all that apply)?

- ☐ Adopting the belief that “gender dysphoria” was the only explanation for the feelings and emotions you were already having (1)
- ☐ Reinterpreting your past feelings and behaviors to be consistent with gender dysphoria or transgender identity (4)
- ☐ The labeling of your feelings and experiences as “gender dysphoria” or “transgender” (5)
- ☐ Considering your past and current feelings and experiences as proof of being transgender (6)
- ☐ The belief that transition would be the solution to your problems (7)
- ☐ None of the above (8)

The term “rapid-onset gender dysphoria” has been used to describe a situation where someone who did not have gender dysphoria during their childhood, appears to suddenly develop gender dysphoria during or after puberty. Does this description fit your experience?

- ○ Yes (1)
- ○ No (4)
- ○ Don't know (5)

End of Block: Research Survey: Becoming Transgender-identified

---

Start of Block: Research Survey: While Transgender-identified

When you were identifying as transgender, did you feel (choose all that apply)?

- ☐ A strong difference between your experienced/expressed gender and your physical body (1)
- ☐ A strong desire to be rid of the body parts/sex characteristics of your natal sex (4)
- ☐ A strong desire to possess the body parts/sex characteristics of the opposite natal sex (5)
- ☐ A strong desire to be the opposite natal sex (6)
- ☐ A strong desire to be treated as the opposite natal sex (7)
- ☐ A strong conviction that you had the typical feelings and reactions of the opposite natal sex (8)
- ☐ None of the above (9)

If you checked any items in the last question, for how long did these feelings last? (For example: strong desire to be opposite natal sex- 1 year; strong desire to be treated as the opposite natal sex- 3 months)

____

____

____

____

____

While you were identifying as transgender, how would you rate your gender dysphoria on a scale of 0-7, with "0" meaning that you don't notice (or barely notice) any distress over your natal sex and "7" meaning that your distress over your natal sex is so severe that it strongly

interferes with your ability to function in your daily life? On a scale from 0-7...

	0 = Barely noticeable 7 = Severe distress
	0 1 2 3 4 5 6 7
Gender dysphoria rating ()	

While you were identifying as transgender, what was your living situation (choose all that apply)?

- ☐ Living with one or more of your parents (1)
- ☐ Living on a college or University campus (4)
- ☐ Living on your own (with or without a roommate) (5)
- ☐ Living with a partner (7)
- ☐ Living with a spouse (8)
- ☐ Other (6)

On a scale of 0-10, please respond to the following questions for the time that you were identifying as transgender.

0 1 2 3 4 5 6 7 8 9 10

Overall, how satisfied were you with life as a whole those days? (0 = Not Satisfied at All, 10 = Completely Satisfied) ()	
In general, how happy or unhappy did you usually feel? (0 = Extremely Unhappy, 10 = Extremely Happy) ()	
In general, how would you have rated your physical health?(0 = Poor, 10 = Excellent) ()	
How would you have rated your overall mental health?(0 = Poor, 10 = Excellent) ()	
Overall, to what extent did you feel the things you did in your life were worthwhile? (0 = Not at All Worthwhile, 10 = Completely Worthwhile) ()	
I understood my purpose in life. (0 = Strongly Disagree, 10 = Strongly Agree) ()	
I always acted to promote good in all circumstances, even in difficult and challenging situations. (0 = Not True of Me, 10 = Completely True of Me) ()	
I was always able to give up some happiness in the present for greater happiness later.(0 = Not True of Me, 10 = Completely True of Me) ()	
I was content with my friendships and relationships. (0 = Strongly Disagree, 10 = Strongly Agree) ()	
My relationships were as satisfying as I wanted them to be. (0 = Strongly Disagree, 10 = Strongly Agree) ()	
How often did you worry about being able to meet normal monthly living expenses? (0 = Worry All of the Time, 10 = Do Not Ever Worry) ()	
How often did you worry about safety, food, or housing? (0 = Worry All of the Time, 10 = Do Not Ever Worry) ()	

Did any members of your in real life/offline friend group desist or detransition before you did?

- ◯ Yes (1)
- ◯ No (4)
- ◯ N/A (6)

Display This Question:

If Did any members of your in real life/offline friend group desist or detransition before you did? = Yes

When someone from your in real life/offline friend group desisted or detransitioned, how did the rest of the friend group react to them? The friend group was...(choose all that apply)?

- ▢ Supportive (3)
- ▢ Neutral (neither supportive or unsupportive) (5)
- ▢ Unsupportive (12)
- ▢ Dismissive or mocking (13)
- ▢ Angry (14)
- ▢ None of the above (15)

Display This Question:

If Did any members of your in real life/offline friend group desist or detransition before you did? = Yes

After someone from your in real life/offline friend group desisted or detransitioned, what was that person's connection (people's connections) with the rest of the friend group (choose all that apply)?

- ☐ They remained in the friend group (1)
- ☐ They left the friend group (it was their own decision) (4)
- ☐ They were excluded from the friend group (8)

Display This Question:

If Did any members of your in real life/offline friend group desist or detransition before you did? = Yes

When someone from your in real life/offline friend group desisted or detransitioned, did it ...(choose all that apply)?

- ☐ Increase your desire to transition (1)
- ☐ Decrease your desire to transition (4)
- ☐ Not have any effect on your desire to transition (5)

Did any members of your online friend group desist or detransition before you did?

- ○ Yes (1)
- ○ No (2)
- ○ N/A (3)

Display This Question:

If Did any members of your online friend group desist or detransition before you did? = Yes

When someone from your online friend group desisted or detransitioned, how did the rest of the friend group react to them? The friend group was...(choose all that apply)?

☐ Supportive (1)

☐ Neutral (neither supportive or unsupportive) (2)

☐ Unsupportive (3)

☐ Dismissive or mocking (4)

☐ Angry (5)

☐ None of the above (6)

Display This Question:

If Did any members of your online friend group desist or detransition before you did? = Yes

After someone from your online friend group desisted or detransitioned, what was that person's connection (people's connections) with the rest of the friend group (choose all that apply)?

☐ They remained in the friend group (5)

☐ They left the friend group (it was their own decision) (6)

☐ They were excluded from the friend group (7)

Display This Question:

If Did any members of your online friend group desist or detransition before you did? = Yes

When someone from your online friend group desisted or detransitioned, did it ...(choose all that apply)?

- ☐ Increase your desire to transition (1)
- ☐ Decrease your desire to transition (2)
- ☐ Not have any effect on your desire to transition (3)

While you were identifying as transgender how did you identify your sexual orientation (choose all that apply)?

- ☐ Asexual (1)
- ☐ Bisexual (4)
- ☐ Demisexual (5)
- ☐ Gay (6)
- ☐ Heterosexual (7)
- ☐ Lesbian (8)
- ☐ Pansexual (9)
- ☐ Queer (10)
- ☐ Other (11)

For all sexual orientations that you checked in the past question, please indicate what age you identified with each label. For example: Heterosexual (until age 12); Gay (12-15); Bisexual (15-25)

____

____

____

____

____

While you were identifying as transgender, what was your sexual attraction to cisgender males versus cisgender females? (attraction to transgender and nonbinary individuals will be asked in the next question)

- ○ Exclusively sexually attracted to males (1)
- ○ Mostly sexually attracted to males (4)
- ○ Somewhat more sexually attracted to males (5)
- ○ Equally sexually attracted to males and females (6)
- ○ Somewhat more sexually attracted to females (7)
- ○ Mostly sexually attracted to females (8)
- ○ Exclusively sexually attracted to females (9)
- ○ Not sexually attracted to males or females (10)

While you were identifying as transgender, how would you describe your sexual attraction to the following (cisgender, transgender and non binary) females and males with 0 meaning "not at all attracted" and 100 meaning "extremely attracted"?

0= Not at all attracted 100= Extremely attracted

0 10 20 30 40 50 60 70 80 90 100

Cisgender women, cisgender girls (natal female sex) ()	
Nonbinary & AFAB (natal female sex) ()	
Transmen, transboys & AFAB (natal female sex) ()	
Cisgender men, cisgender boys (natal male sex) ()	
Nonbinary & AMAB (natal male sex) ()	
Transwomen, transgirls & AMAB (natal male sex) ()	

While you were identifying as transgender, did you have romantic or sexual relationships with people of the following genders and sexes (choose all that apply)?

	Yes (1)	No (2)
Cisgender women, cisgender girls (natal female sex) (1)	○	○
Nonbinary & AFAB (natal female sex) (4)	○	○
Transmen, transboys & AFAB (natal female sex) (5)	○	○
Cisgender men, cisgender boys (natal male sex) (6)	○	○
Nonbinary & AMAB (natal male sex) (7)	○	○
Transwomen, transgirls & AMAB (natal male sex) (8)	○	○

Which of the following steps did you take while identifying as transgender?

- ☐ Asked to be called by a different name (1)
- ☐ Asked to be referred to by different pronouns (4)
- ☐ Changed hairstyle, clothing, or makeup to be more in line with the target gender (5)
- ☐ Used a binder (6)
- ☐ Used breast forms (7)
- ☐ Used a gaff (8)
- ☐ Used a prosthetic penis (9)
- ☐ None of the above (10)

Which of the following medications have you taken for the purpose of transitioning (or trying to alleviate gender dysphoria)?

- ☐ Puberty blockers (1)
- ☐ Cross-sex hormones- testosterone (4)
- ☐ Cross-sex hormones- estrogen (5)
- ☐ Anti-androgens (6)
- ☐ None of the above (7)

If you have taken puberty blockers, cross-sex hormones, or anti-androgens, please write how long you took each one.

____

____

____

____

____

Which, if any, type of surgery did you have for the purpose of transition (or trying to alleviate gender dysphoria) (choose all that apply)?

- ☐ Breast, chest, or top surgery to remove breasts (1)
- ☐ Breast surgery to augment breasts (4)
- ☐ Surgery to remove uterus (5)
- ☐ Surgery to remove ovaries (6)
- ☐ Surgery to remove testes (7)
- ☐ Surgery to remove penis (8)
- ☐ Surgery to create a penis (9)
- ☐ Surgery to create a vagina (10)
- ☐ None of the above (11)

Did you see a doctor, mental health provider, or other clinician in order to obtain cross-sex hormones?

- Yes (1)
- No (4)

End of Block: Research Survey: While Transgender-identified

Start of Block: Research Survey: Informed Consent for medical transition

Display This Question:

If Did you see a doctor, mental health provider, or other clinician in order to obtain cross-sex hor... = Yes

What type of doctor or clinician did you see in order to obtain cross-sex hormones?

- Primary care physician (such as an internist, pediatrician, family medicine doctor) (1)
- Psychiatrist who treats children and adolescents (4)
- Psychiatrist who treats adults (5)
- Social worker (6)
- Nurse practitioner (7)
- Endocrinologist (8)
- Don’t know (9)
- None of the above (10)
- Other (12)

Display This Question:

If Did you see a doctor, mental health provider, or other clinician in order to obtain cross-sex hor... = Yes

What type of practice or organization did you visit to obtain cross-sex hormones from (choose all that apply)?

- ☐ A clinic specializing in gender issues (1)
- ☐ A general health clinic (4)
- ☐ A Planned Parenthood (5)
- ☐ A private practice (6)
- ☐ A private gender clinic (8)
- ☐ Other (9)

Display This Question:

If Did you see a doctor, mental health provider, or other clinician in order to obtain cross-sex hor... = Yes

How old were you when you went to see a doctor or clinician to obtain cross-sex hormones?

____

Display This Question:

If Did you see a doctor, mental health provider, or other clinician in order to obtain cross-sex hor... = Yes

Was the practice or organization where you visited to obtain cross-sex hormones one that specifically used the "informed consent" model of care?

- Yes (1)
- No (4)
- Don't know (5)

Display This Question:

If Did you see a doctor, mental health provider, or other clinician in order to obtain cross-sex hor... = Yes

Did you receive cross-sex hormones (or a prescription for cross-sex hormones)?

- Yes (1)
- No (2)

Display This Question:

If Did you receive cross-sex hormones (or a prescription for cross-sex hormones)? = No

Please indicate the reasons that you did not receive cross-sex hormones (or a prescription for cross-sex hormones). (choose all that apply)

- ☐ The doctor or clinician did not prescribe cross-sex hormones for you (1)
- ☐ You decided that you did not want cross-sex hormones (2)
- ☐ Your parent(s) did not permit you to receive a prescription for cross-sex hormones (4)

Display This Question:

If Did you receive cross-sex hormones (or a prescription for cross-sex hormones)? = Yes

How many times did you visit the clinician before receiving a prescription for cross-sex hormones?

____

Display This Question:

If Did you receive cross-sex hormones (or a prescription for cross-sex hormones)? = Yes

If you received cross-sex hormones after one visit, how long was that one visit?

- ◯ Less than 15 minutes (1)
- ◯ 15 - 30 minutes (4)
- ◯ More than 30 minutes but less than an hour (5)
- ◯ One hour or more but less than 2 hours (6)
- ◯ Two hours or more but less than three hours (7)
- ◯ Three hours or longer (8)
- ◯ N/A (9)

Display This Question:

If Did you receive cross-sex hormones (or a prescription for cross-sex hormones)? = Yes

Before receiving your prescription for cross-sex hormones, were you evaluated by a mental health professional (psychologist, psychiatrist, licensed clinical social worker)?

- ◯ Yes (1)
- ◯ No (4)
- ◯ Don’t know (5)

Display This Question:

If Did you receive cross-sex hormones (or a prescription for cross-sex hormones)? = Yes

Before providing a prescription for cross-sex hormones, did your clinician ask you about the symptoms you were having?

- Yes (1)
- No (4)
- Don't know (5)

Display This Question:

If Did you receive cross-sex hormones (or a prescription for cross-sex hormones)? = Yes

Before providing a prescription for cross-sex hormones, did the clinician ask you why you thought cross-sex hormones would be helpful to you?

- Yes (1)
- No (4)
- Don't know (5)

Display This Question:

If Did you receive cross-sex hormones (or a prescription for cross-sex hormones)? = Yes

Before providing a prescription for cross-sex hormones, did the clinician ask in detail about (choose all that apply)?

- ▢ The nature and development of your gender dysphoria during your childhood and teen years (1)
- ▢ Whether your gender dysphoria started during your childhood (early-onset) or during or after puberty (late-onset) (4)
- ▢ Your understanding that stigma related to gender nonconformity can have mental health effects (5)
- ▢ Your sexual identity, sexual orientation, and behaviors (6)
- ▢ Previous traumatic experiences (7)
- ▢ Whether you are associated with or engaging in kink experiences or communities (8)
- ▢ What your expectations are for transition (9)
- ▢ Whether your expectations for transition are realistic (10)
- ▢ What kind of support you had from family and friends (11)
- ▢ None of the above (12)

Display This Question:

If Did you receive cross-sex hormones (or a prescription for cross-sex hormones)? = Yes

Before providing a prescription for cross-sex hormones, did your clinician ask you questions to explore whether there might be specific causes for your symptoms of gender dysphoria?

- ○ Yes (1)
- ○ No (4)
- ○ Don’t know (5)

Display This Question:

If Did you receive cross-sex hormones (or a prescription for cross-sex hormones)? = Yes

Before providing a prescription for cross-sex hormones, did the clinician ask you questions to explore whether you had any condition other than gender dysphoria that needed to be addressed?

- ○ Yes (1)
- ○ No (4)
- ○ Don’t know (5)

Display This Question:

If Did you receive cross-sex hormones (or a prescription for cross-sex hormones)? = Yes

Before providing a prescription for cross-sex hormones which, if any, of the following conditions did your clinician ask you about?

☐ Addiction (or overuse) of any substance (alcohol, opiates, painkillers, marijuana, cocaine, etc) (1)

☐ Anxiety (4)

☐ Attention Deficit Hyperactivity Disorder (ADHD) or ADD (5)

☐ Autism Spectrum Disorders (ASD) (6)

☐ Body dysmorphia (7)

☐ Bipolar disorder (8)

☐ Borderline personality disorder (9)

☐ Depression (10)

☐ Drug use (including but not limited to marijuana, cocaine, ecstasy, etc) (11)

☐ Eating disorders (such as anorexia, bulimia) (12)

☐ History of abuse or neglect (13)

☐ Morbid obesity or past history of obesity (14)

☐ Obsessive Compulsive Disorder (OCD) (15)

☐ Personality disorders (16)

☐ Post-traumatic Stress Disorder (PTSD) (17)

☐ Self-harm or Non-Suicidal Self Injury (NSSI) (18)

☐ Sexual trauma (including rape, sexual abuse, attempted rape) (19)

☐ Hypomanic or manic experiences (20)

☐ None of the above (21)

☐ Don't know (22)

Display This Question:

If Did you receive cross-sex hormones (or a prescription for cross-sex hormones)? = Yes

If any of the conditions in the previous question were present, did the clinician make a plan for further evaluation or treatment of these conditions?

○ Yes (1)

○ No (4)

○ N/A (5)

Display This Question:

If Did you receive cross-sex hormones (or a prescription for cross-sex hormones)? = Yes

Before providing a prescription for cross-sex hormones, did your clinician ask you questions to explore if you had any condition that might impair your ability to make an informed decision about whether to proceed with medical transition?

○ Yes (1)

○ No (4)

○ Don't know (5)

Display This Question:

If Did you receive cross-sex hormones (or a prescription for cross-sex hormones)? = Yes

Before you received a prescription for cross-sex hormones, did the clinician inform you about the risks of medical transition (cross-sex hormones) ?

- Yes (1)
- No (4)
- Don't know (5)

Display This Question:

If Did you receive cross-sex hormones (or a prescription for cross-sex hormones)? = Yes

Do you believe that the information that the clinician gave you about the risks of medical transition (cross-sex hormones) was adequate?

- Yes (1)
- No (4)
- Don't know (5)
- N/A (6)

Display This Question:

If Did you receive cross-sex hormones (or a prescription for cross-sex hormones)? = Yes

What risks for medical transition (cross-sex hormones) did the clinician tell you about?

____

____

____

____

____

Display This Question:

If Did you receive cross-sex hormones (or a prescription for cross-sex hormones)? = Yes

What risks for medical transition (cross-sex hormones) do you wish the clinician told you about?

____

____

____

____

____

Display This Question:

If Did you receive cross-sex hormones (or a prescription for cross-sex hormones)? = Yes

Before you received a prescription for cross-sex hormones, did the clinician inform you about the benefits of medical transition (cross-sex hormones) ?

- ○ Yes (1)
- ○ No (4)
- ○ Don't know (5)

Display This Question:

If Did you receive cross-sex hormones (or a prescription for cross-sex hormones)? = Yes

Do you believe that the information that the clinician gave you about the benefits of medical transition (cross-sex hormones) was adequate?

- ○ Yes (1)
- ○ No (4)
- ○ Don't know (5)
- ○ N/A (6)

Display This Question:

If Did you receive cross-sex hormones (or a prescription for cross-sex hormones)? = Yes

What benefits of medical transition (cross-sex hormones) did the clinician tell you about?

____

____

____

____

____

Display This Question:

If Did you receive cross-sex hormones (or a prescription for cross-sex hormones)? = Yes

What benefits of medical transition (cross-sex hormones) do you wish the clinician told you about?

____

____

____

____

____

Display This Question:

If Did you receive cross-sex hormones (or a prescription for cross-sex hormones)? = Yes

Please indicate which of the following potential benefits of medical transition (cross-sex hormones) were discussed with you (choose all that apply)

- ☐ Improved mental health (1)
- ☐ Improved physical health (4)
- ☐ Increased confidence (5)
- ☐ Decreased dysphoria (9)
- ☐ Eliminated dysphoria (10)
- ☐ That you would be living your authentic life (6)
- ☐ None of the above (7)

Display This Question:

If Did you receive cross-sex hormones (or a prescription for cross-sex hormones)? = Yes

Please indicate which of the following potential risks of medical transition (cross-sex hormones) were discussed with you (choose all that apply).

- ☐ Depression (1)
- ☐ Anxiety (4)
- ☐ Self-harm (5)
- ☐ Suicidal thoughts or ideation (6)
- ☐ Worsening of dysphoria symptoms (7)
- ☐ No improvement in dysphoria symptoms (8)
- ☐ Dissatisfaction with the physical changes that occur from transition (9)
- ☐ Regret of the decision to transition (10)
- ☐ Detransition (11)
- ☐ Feeling “inauthentic” after transitioning (12)
- ☐ Possible loss of bone strength (osteoporosis) (13)
- ☐ Unknown long-term medical risks (14)
- ☐ Blood clots in legs or lungs (15)
- ☐ Heart attacks, stroke, high blood pressure (16)
- ☐ Shortened life expectancy (17)
- ☐ Infertility or difficulty becoming (or getting someone) pregnant (18)

☐ Decrease or loss of sexual pleasure (including difficulty in reaching orgasm, difficulty getting or maintaining an erection, decrease in sex drive, pain with intercourse) (19)

☐ Increased sex drive (20)

☐ Vaginal dryness (21)

☐ Urinary incontinence (uncontrolled loss of urine/wetting of self) (22)

☐ Needing to have surgery because of the hormone treatments (23)

☐ Strained relationships (or loss of relationships) with family and current friends (24)

☐ Exchange of existing friends for friends from the transgender community (25)

☐ Difficulty in dating (26)

☐ Reduced number of people willing to have a romantic, intimate or sexual relationships (27)

☐ Social discrimination (28)

☐ Distraction from working on academic, career, and other goals. (29)

☐ Outsider status (30)

☐ Risks from discontinuing cross-sex hormones (31)

☐ None of the above (32)

☐ Don't know (33)

Display This Question:

If Did you receive cross-sex hormones (or a prescription for cross-sex hormones)? = Yes

Did the clinician tell you that the benefits of medical transition (cross-sex hormones) would outweigh the risks for you?

- ○ Yes (1)
- ○ No (4)
- ○ Don’t know (5)

Display This Question:

If Did you receive cross-sex hormones (or a prescription for cross-sex hormones)? = Yes

Did the clinician tell you that for teens and young adults whose gender dysphoria began during or after puberty, it is unknown how long their gender dysphoria is likely to last?

- ○ Yes (1)
- ○ No (4)
- ○ Don’t know (5)

Display This Question:

If Did you receive cross-sex hormones (or a prescription for cross-sex hormones)? = Yes

Did the clinician tell you that there are no long-term studies about natal female teens and young adults whose gender dysphoria began during or after puberty?

- ○ Yes (1)
- ○ No (4)
- ○ Don’t know (5)

Display This Question:

If Did you receive cross-sex hormones (or a prescription for cross-sex hormones)? = Yes

Did the clinician tell you that for teens and young adults whose gender dysphoria began during or after puberty, the risks, benefits and outcomes for medical transition (cross-sex hormones) are unknown?

- ○ Yes (1)
- ○ No (4)
- ○ Don’t know (5)

Display This Question:

If Did you receive cross-sex hormones (or a prescription for cross-sex hormones)? = Yes

Before providing a prescription for cross-sex hormones, did your clinician tell you about any alternatives to medical transition (including the possibility of not taking cross-sex hormones/not medically transitioning)?

- ○ Yes (1)
- ○ No (4)
- ○ Don’t know (5)

Display This Question:

If Did you receive cross-sex hormones (or a prescription for cross-sex hormones)? = Yes

Before providing a prescription for cross-sex hormones, did your clinician tell you about the risks and benefits of any alternatives to medical transition (including the risks and benefits of not taking cross-sex hormones/not medically transitioning)?

- ○ Yes (1)
- ○ No (4)
- ○ Don't know (5)

Display This Question:

If Did you receive cross-sex hormones (or a prescription for cross-sex hormones)? = Yes

Do you believe that the information you received about the alternatives to medical transition was adequate?

○ Yes (1)

○ No (4)

○ Don't know (5)

○ N/A (6)

Display This Question:

If Did you receive cross-sex hormones (or a prescription for cross-sex hormones)? = Yes

What alternatives to medical transition (cross-sex hormones) did the clinician tell you about?

____

____

____

____

____

Display This Question:

If Did you receive cross-sex hormones (or a prescription for cross-sex hormones)? = Yes

What alternatives to medical transition (cross-sex hormones) do you wish the clinician told you about?

____

____

____

____

____

Display This Question:

If Did you receive cross-sex hormones (or a prescription for cross-sex hormones)? = Yes

Were you offered any of the following prior to receiving a prescription for cross-sex hormones?

- ☐ Extended evaluation to determine if transition would be helpful to you (1)
- ☐ Psychotherapy to work on existing mental health issues or traumas (4)
- ☐ Psychotherapy to consider your motives in greater detail (5)
- ☐ Time to mature and gain life experience before starting transition (6)
- ☐ A family meeting to clarify their feelings about this (7)
- ☐ None of the above (8)

Display This Question:

If Did you receive cross-sex hormones (or a prescription for cross-sex hormones)? = Yes

Did your clinician tell you that…?

- ○ Medical transition (cross-sex hormones) would decrease your risk of suicide (1)
- ○ Medical transition (cross-sex hormones) would increase your risk of suicide (4)
- ○ There isn't enough research to know if medical transition (cross-sex hormones) decreases, increases, or fails to change risk of suicide. (5)
- ○ Suicide risk was not mentioned by my clinician (6)

Display This Question:

If Did you receive cross-sex hormones (or a prescription for cross-sex hormones)? = Yes

At the time you received your prescription, did your clinician ask you to schedule a follow-up visit?

- Yes (1)
- No (4)
- Don't Know (5)

Display This Question:

If Did you receive cross-sex hormones (or a prescription for cross-sex hormones)? = Yes

If you were scheduled for a follow-up visit, when were you supposed to return to the clinic?

- In less than 3 months (1)
- Between 3 months and 6 months (4)
- Between 7 and 12 months (5)
- No follow-up visit was scheduled (6)

Display This Question:

If Did you receive cross-sex hormones (or a prescription for cross-sex hormones)? = Yes

Did you inform the clinician who facilitated your transition that you have desisted or detransitioned?

- Yes (1)
- No (4)

Display This Question:

If Did you receive cross-sex hormones (or a prescription for cross-sex hormones)? = Yes

If there was a reason why you did or did not contact the clinician to let them know you desisted or detransitioned, please describe.

____

____

____

____

____

Display This Question:

If Did you receive cross-sex hormones (or a prescription for cross-sex hormones)? = Yes

Overall, were you satisfied with the medical care you received?

- ○ Yes (1)
- ○ No (4)
- ○ Don't know (5)

Display This Question:

If Overall, were you satisfied with the medical care you received? = No

If you were dissatisfied with the quality of medical care that you received, have you...?

- ▢ Submitted a complaint to the medical licensing board (1)
- ▢ Contacted an attorney for legal advice about a lawsuit (4)
- ▢ None of the above (5)

End of Block: Research Survey: Informed Consent for medical transition

Start of Block: Research Survey: Desisting and After Desistance

In your own words, do you think anything contributed to why you stopped identifying as transgender? If so, what?

____

____

____

____

____

When you stopped identifying as transgender, how important were each of the following factors in that process?

	Not applicable (N/A) (1)	Not at all important (7)	Somewhat important (8)	Moderately important (9)	Very important (10)	Extremely important (11)
Transphobia or discrimination experienced while identifying as transgender (1)	○	○	○	○	○	○
Pressure from family (4)	○	○	○	○	○	○
Feeling that identifying as transgender served a purpose at the time, but was not needed anymore (5)	○	○	○	○	○	○
Peer pressure (6)	○	○	○	○	○	○
Your own thought processes (7)	○	○	○	○	○	○
Your personal definition of “male” and “female” changed and you now felt comfortable identifying as your natal sex (8)	○	○	○	○	○	○
Worsened mental health while identifying as transgender (9)	○	○	○	○	○	○

Lack of improvement in mental health while identifying as transgender (10)	o	o	o	o	o	o
Feeling that “transgender” did not fit who you were anymore (11)	o	o	o	o	o	o
Difficulty in finding someone to have a dating, romantic or sexual relationship with (12)	o	o	o	o	o	o
Missing your life from before coming out or before transition (13)	o	o	o	o	o	o
Wishing you could go back to being cisgender (14)	o	o	o	o	o	o
Dissatisfaction with the physical changes that occurred with transition (15)	o	o	o	o	o	o
Discovering that the gender dysphoria was caused by something specific like a trauma or mental health condition (16)	o	o	o	o	o	o

Feeling that the causes for your gender dysphoria were more complicated that you previously thought they were (17)	○	○	○	○	○	○
Religion or religious beliefs (18)	○	○	○	○	○	○
Your expectations about what things in your life would improve did not match up in reality (19)	○	○	○	○	○	○
The strong emotions you were having that lead you to identify as transgender resolved (20)	○	○	○	○	○	○
Feeling uncomfortable with the transgender community (21)	○	○	○	○	○	○
There was a change in your political or philosophical views (22)	○	○	○	○	○	○

Now that you are no longer identifying as transgender, do you feel (choose all that apply)?

- ☐ A strong difference between your experienced/expressed gender and your physical body (1)
- ☐ A strong desire to be rid of the body parts/sex characteristics of your natal sex (4)
- ☐ A strong desire to possess the body parts/sex characteristics of the opposite natal sex (5)
- ☐ A strong desire to be the opposite natal sex (6)
- ☐ A strong desire to be treated as the opposite natal sex (7)
- ☐ A strong conviction that you had the typical feelings and reactions of the opposite natal sex (8)
- ☐ None of the above (9)

If you checked any of the items in the last question, for how long did you feel this way? (example: desire to be the opposite natal sex- 8 months, desire to be treated- 1 year)

____

____

____

____

____

Now that you are not identifying as transgender, how would you rate your gender dysphoria on a scale of 0-7, with "0" meaning that you don't notice (or barely notice) any distress over your natal sex and "7" meaning that your distress over your natal sex is so severe that it strongly interferes with your ability to function in your daily life? On a scale from 0-7 …

0 = Barely noticeable 7 = Severe distress

	0	1	2	3	4	5	6	7
Gender dysphoria rating ()								

What is your current living situation (choose all that apply)?

- ☐ Living with one or more of your parents (1)
- ☐ Living on a college or University campus (4)
- ☐ Living on your own (with or without a roommate) (5)
- ☐ Living with a partner (7)
- ☐ Living with a spouse (8)
- ☐ Other (6)

On a scale of 0-10, please respond to the following questions for the present time.

0 1 2 3 4 5 6 7 8 9 10

Overall, how satisfied are you with life as a whole these days? (0 = Not Satisfied at All, 10 = Completely Satisfied) ()	
In general, how happy or unhappy do you usually feel? (0 = Extremely Unhappy, 10 = Extremely Happy) ()	
In general, how would you rate your physical health?(0 = Poor, 10 = Excellent) ()	
How would you rate your overall mental health?(0 = Poor, 10 = Excellent) ()	
Overall, to what extent do you feel the things you do in your life are worthwhile? (0 = Not at All Worthwhile, 10 = Completely Worthwhile) ()	
I understand my purpose in life. (0 = Strongly Disagree, 10 = Strongly Agree) ()	
I always act to promote good in all circumstances, even in difficult and challenging situations. (0 = Not True of Me, 10 = Completely True of Me) ()	
I am always able to give up some happiness now for greater happiness later.(0 = Not True of Me, 10 = Completely True of Me) ()	
I am content with my friendships and relationships. (0 = Strongly Disagree, 10 = Strongly Agree) ()	
My relationships are as satisfying as I would want them to be. (0 = Strongly Disagree, 10 = Strongly Agree) ()	
How often do you worry about being able to meet normal monthly living expenses? (0 = Worry All of the Time, 10 = Do Not Ever Worry) ()	
How often do you worry about safety, food, or housing? (0 = Worry All of the Time, 10 = Do Not Ever Worry) ()	

Have you EVER been diagnosed with any of the following (choose all that apply)?

- [ ] Anxiety (1)
- [ ] Attention Deficit Hyperactivity Disorder (ADHD) (4)
- [ ] Autism Spectrum Disorders (5)
- [ ] Bipolar Disorder (6)
- [ ] Borderline Personality Disorder (7)
- [ ] Depression (8)
- [ ] Eating Disorders (9)
- [ ] History of pulling out hairs (example eyelashes, eyebrows) when anxious (10)
- [ ] Obsessive Compulsive Disorder (11)
- [ ] Post-Traumatic Stress Disorder (PTSD) (12)
- [ ] Schizophrenia or Psychosis (13)
- [ ] Selective mutism (14)
- [ ] Tourette's (15)
- [ ] None of the above (16)
- [ ] Other (17)

If you checked any items in the previous question, please indicate the ages that you had the condition.

____

____

____

____

____

When, if ever, have you engaged in self harm or Non-suicidal self-injury (NSSI) such as cutting, burning, or picking (choose all that apply)?

- ▢ Never (1)
- ▢ Before identifying as transgender (4)
- ▢ While identifying as transgender (5)
- ▢ After you stopped identifying as transgender (6)

Now that you are not identifying as transgender, how do you identify your sexual orientation (choose all that apply)?

- ☐ Asexual (1)
- ☐ Bisexual (4)
- ☐ Demisexual (5)
- ☐ Gay (6)
- ☐ Heterosexual (7)
- ☐ Lesbian (8)
- ☐ Pansexual (9)
- ☐ Queer (10)
- ☐ Other (11)

For all sexual orientations that you checked in the past question, please indicate what age you identified with each label. For example: Heterosexual male (until age 12); Gay male (12-15); Bisexual male (15-25)

____

____

____

____

____

Now that you are not identifying as transgender, what is your sexual attraction to cisgender males versus cisgender females? (attraction to transgender and nonbinary individuals will be asked in the next question)

- ○ Exclusively sexually attracted to males (1)
- ○ Mostly sexually attracted to males (4)
- ○ Somewhat more sexually attracted to males (5)
- ○ Equally sexually attracted to males and females (6)
- ○ Somewhat more sexually attracted to females (7)
- ○ Mostly sexually attracted to females (8)
- ○ Exclusively sexually attracted to females (9)
- ○ Not sexually attracted to males or females (10)

Now that you are not identifying as transgender how would you describe your sexual attraction to the following (cisgender, transgender and non binary) females and males with 0 meaning "not at all attracted" and 100 meaning "extremely attracted"?

	0= Not at all attracted 100= Extremely attracted<br>0 10 20 30 40 50 60 70 80 90 100
Cisgender women, cisgender girls (natal female sex) ()	
Nonbinary & AFAB (natal female sex) ()	
Transmen, transboys & AFAB (natal female sex) ()	
Cisgender men, cisgender boys (natal male sex) ()	
Nonbinary & AMAB (natal male sex) ()	
Transwomen, transgirls & AMAB (natal male sex) ()	

Since you have stopped identifying as transgender, did you have romantic or sexual relationships with people of the following genders and sexes (choose all that apply)?

	Yes (1)	No (2)
Cisgender women, cisgender girls (natal female sex) (1)	○	○
Non-binary & AFAB (natal female sex) (4)	○	○
Transmen, transboys & AFAB (natal female sex) (5)	○	○
Cisgender men, cisgender boys (natal male sex) (6)	○	○
Non-binary & AMAB (natal male sex) (7)	○	○

Please check all the gender and sexual identities that you have ever used as a label for yourself for at least one month?

- ☐ Agender (1)
- ☐ Asexual (4)
- ☐ Bisexual (5)
- ☐ Demisexual (6)
- ☐ Gay (7)
- ☐ Genderfluid (8)
- ☐ Genderqueer (9)
- ☐ Homosexual (10)
- ☐ Lesbian (11)
- ☐ Non-binary (12)
- ☐ Pansexual (13)
- ☐ Queer (14)
- ☐ Other (15)

If you answered more than one of the options in the last question, please describe the order in which you identified as each label

____

____

____

____

____

Please answer the following for the past six months.

	Never (1)	Rarely (6)	Sometimes (7)	Often (8)	Very often (9)
How often did you go online to look up an answer to a question you had? (1)	o	o	o	o	o
How often did you find information online that was useful and accurate? (4)	o	o	o	o	o
How often did you go online to seek support? (5)	o	o	o	o	o
How often did you get the support you were looking for online? (6)	o	o	o	o	o
How often did you make friends on the internet that you still have today? (7)	o	o	o	o	o

Please answer the following for the past six-months.

	Never (1)	Rarely (6)	Sometimes (7)	Often (8)	Very often (9)
How often did you choose to socialize online instead of in-person? (1)	○	○	○	○	○
How often did you have problems with face to face communication due to your internet use? (4)	○	○	○	○	○
How often did you experience increased social anxiety due to your internet use? (5)	○	○	○	○	○
How often did you fail to make real-life relationships because of the internet? (6)	○	○	○	○	○
How often did you skip out on social events to spend time online? (7)	○	○	○	○	○
How often did your offline relationships suffer due to your internet use? (8)	○	○	○	○	○

How often did you feel irritated when you were not able to use the internet? (9)	○	○	○	○	○
How often did you feel angry because you were away from the internet? (10)	○	○	○	○	○
How often did you feel anxious because you were away from the internet? (11)	○	○	○	○	○
How often did you feel vulnerable when the internet wasn't available? (12)	○	○	○	○	○
How often did you experience feelings of withdrawal from not using the internet? (13)	○	○	○	○	○
How often did you put internet use in front of important, everyday activities? (14)	○	○	○	○	○
How often did you avoid other activities in order to stay online? (15)	○	○	○	○	○

How often did you neglect your responsibilities because of the internet? (16)	○	○	○	○	○
How often did you lose motivation to do other things that need to get done because of the internet? (17)	○	○	○	○	○
How often did you lose sleep due to nighttime internet use? (18)	○	○	○	○	○
How often did time on the internet negatively affect your schoolwork? (19)	○	○	○	○	○
How often did you feel that you used the internet excessively? (20)	○	○	○	○	○

At which point(s) in your life did you feel that you were most true to yourself and authentic (choose all that apply)?

- ☐ Before you identified as transgender (1)
- ☐ While you were identifying as transgender (4)
- ☐ Now that you are not identifying as transgender (5)
- ☐ None of the above (6)

How likely do you think it is that you will identify as transgender sometime in the future?

- ○ Extremely likely (1)
- ○ Very likely (4)
- ○ Moderately likely (5)
- ○ Somewhat unlikely (6)
- ○ Not at all likely (7)

If you could go back in time to before you started to identify as transgender, what would you say to your younger self?

____

____

____

____

____

If you would like, please add anything about your experience that you think will be helpful.

____

____

____

____

____

This survey included questions about trauma, self-harm, and mental health. **These assessments will not be reviewed immediately**. Everyone participating in this research study will receive a list of contacts if they want to speak with someone about any health concerns or abuse. Because this survey is anonymous, there is no way to contact you for follow-up regarding your answers. Please use the following list of contacts and resources if needed: The National Alliance on Mental Illness (NAMI): https://www.nami.org/Find-Support; helpline: 1-800-950-6264; and Crisis textline: 741741 The National Sexual Assault Hotline: https://www.rainn.org/about-national-sexual-assault-telephone-hotline; helpline: 1-800-656-4673 The National Suicide Prevention Lifeline: https://suicidepreventionlifeline.org; helpline: 1-800-273-8255 Substance Abuse and Mental Health Services Association: https://www.samhsa.gov/find-help/national-helpline; helpline: 1-800-662-4357

End of Block: Research Survey: Desisting and After Desistance

---
